# Nicotine suppresses crystalline silica‐induced astrocyte activation and neuronal death by inhibiting NF‐κB in the mouse hippocampus

**DOI:** 10.1111/cns.14508

**Published:** 2023-10-21

**Authors:** Hangbing Cao, Bing Li, Min Mu, Shanshan Li, Haoming Chen, Huihui Tao, Wenyang Wang, Yuanjie Zou, Yehong Zhao, Yang Liu, Xinrong Tao

**Affiliations:** ^1^ Key Laboratory of Industrial Dust Control and Occupational Health of the Ministry of Education Anhui University of Science and Technology Huainan China; ^2^ Key Laboratory of Industrial Dust Deep Reduction and Occupational Health and Safety of Anhui Higher Education Institutes Anhui University of Science and Technology Huainan China; ^3^ Anhui Province Engineering Laboratory of Occupational Health and Safety Anhui University of Science and Technology Huainan China; ^4^ School of Medicine, Department of Medical Frontier Experimental Center Anhui University of Science and Technology Huainan China; ^5^ School of Pharmacy Bengbu Medical College Bengbu China

**Keywords:** astrocyte activation, crystalline silica, neuroinflammation, nicotine

## Abstract

**Aims:**

Exposure to crystalline silica (CS) in occupational settings induces chronic inflammation in the respiratory system and, potentially, the brain. Some workers are frequently concurrently exposed to both CS and nicotine. Here, we explored the impact of nicotine on CS‐induced neuroinflammation in the mouse hippocampus.

**Methods:**

In this study, we established double‐exposed models of CS and nicotine in C57BL/6 mice. To assess depression‐like behavior, experiments were conducted at 3, 6, and 9 weeks. Serum inflammatory factors were analyzed by ELISA. Hippocampus was collected for RNA sequencing analysis and examining the gene expression patterns linked to inflammation and cell death. Microglia and astrocyte activation and hippocampal neuronal death were assessed using immunohistochemistry and immunofluorescence staining. Western blotting was used to analyze the NF‐κB expression level.

**Results:**

Mice exposed to CS for 3 weeks showed signs of depression. This was accompanied by elevated IL‐6 in blood, destruction of the blood–brain barrier, and activation of astrocytes caused by an increased NF‐κB expression in the CA1 area of the hippocampus. The elevated levels of astrocyte‐derived Lcn2 and upregulated genes related to inflammation led to higher neuronal mortality. Moreover, nicotine mitigated the NF‐κB expression, astrocyte activation, and neuronal death, thereby ameliorating the associated symptoms.

**Conclusion:**

Silica exposure induces neuroinflammation and neuronal death in the mouse hippocampal CA1 region and depressive behavior. However, nicotine inhibits CS‐induced neuroinflammation and neuronal apoptosis, alleviating depressive‐like behaviors in mice.

## Graphical abstract

Pattern diagram illustrating the alleviation of depression induced by crystalline silica through nicotine intervention. Upon exposure to CS, the blood–brain barrier (BBB) in the hippocampus undergoes damage, leading to activation of astrocytes and microglia. The activated astrocytes facilitate the secretion of Lcn2 via the NF‐κB pathway, ultimately resulting in neuronal death. However, nicotine mitigates the activation of astrocytes and microglia while safeguarding neurons against cell death.

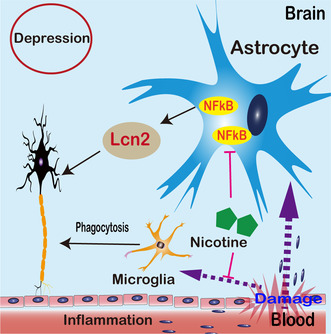

## 1 INTRODUCTION

Exposure to crystalline silica (CS) is a prevalent issue faced by workers in industries such as mining, road building, and quarrying, and emotional disturbances in workers exposed to dust are a public health concern. Epidemiological studies indicate high rates of depression‐like mood among workers following CS exposure.^1^, ^2^ In a survey of 665 male workers who were frequently exposed to particulate matter, 294 (44.2%) exhibited depressive symptoms while 171 (25.7%) showed both anxiety and depressive symptoms.^3^ Furthermore, more than 50% of workers in each occupational exposure group were smokers.^3^, ^4^ Exposure to nicotine in cigarettes has the potential to provide immediate relief to workers who experience negative emotions due to their exposure to CS. Nevertheless, the exact mechanisms by which CS exposure triggers mood disorders and the impact of nicotine on mood regulation in individuals who are occupationally exposed to CS are not currently well understood.

Transient neuroinflammatory signaling plays a protective role during development and tissue repair following injury, while chronic neuroinflammation is associated with the progression of neurodegenerative diseases such as Alzheimer's disease, Parkinson's disease, amyotrophic lateral sclerosis, and multiple sclerosis.^5^ Chronic CS exposure has been shown to cause inflammation of the central nervous system (CNS), leading to depression and a decline in cognitive abilities.^6^, ^7^ There is a growing understanding that occupational exposure to dust can impact brain function in various ways, with effects on oxidative stress, the inflammatory response, and neurotransmitters.^8^ Prior research has demonstrated the significance of hippocampal regions in regulating mood and cognitive functioning. Reduced neuronal activity within the CA1 subfield of the hippocampus could potentially underlie the manifestation of depressive behaviors.^9^ Notably, the CA1 region, which contains a high density of neurons and requires substantial energy and blood supply, is particularly vulnerable to external damage due to the low density of corresponding blood vessels and relatively low energy supply.^10^, ^11^ Simultaneously, hippocampal neurons can modulate affective states and behavioral responses through synaptic connections with neighboring brain areas, including the prefrontal cortex and amygdala.^12^ Disruption of the blood–brain barrier (BBB) facilitates the entry of exogenous substances into the brain, triggering neuroinflammation. Substantial evidence implicates an amplified neuroinflammatory response and neuronal apoptosis in the pathogenesis of depression.^13^ In addition, various anti‐inflammatory agents have been shown to mitigate neuroinflammatory responses and ameliorate depressive symptoms, partially restoring the homeostasis of the nervous system.^14^ Thus, preservation of the BBB and attenuation of the neuroinflammatory response hold tremendous therapeutic potential for nervous system‐related disorders.

Evidence suggests that astrocyte activation and neuronal death play crucial roles in the development of depression‐like emotions.^15^ Inflammation triggers astrocyte activation and proliferation, which lead to the induction of various biological effects, including cytokine production and oxidative stress. Through the secretion of cytokines, neurotransmitters and other signaling molecules, astrocytes can modulate neuronal activity and synaptic plasticity.^16^ Clinical studies have further shown that Lcn2 expression is significantly elevated in hippocampal injury and in ischemic and neurodegenerative conditions such as Alzheimer's disease.^17^ Specifically, activated astrocytes can produce numerous cytokines and neurotransmitters, such as tumor necrosis factor‐alpha (TNF‐α), interleukin 1β (IL‐1β), glutamate, and dopamine, which can significantly influence neuronal activity and synaptic plasticity.^18^ In addition, activated astrocytes secrete reactive nitrogen species, oxygen radicals, and lipid peroxides, among other molecules, inducing oxidative stress and inflammatory responses that can lead to neuronal damage and death. Recent studies have revealed that activated astrocytes can secrete Lcn2, a secretory member of the lipid transporter family, and cause the deposition of iron ions in neurons by binding to neuronal receptors, culminating in neuronal death.^19^

Nicotine, a significant constituent of cigarette smoke, can alleviate depression.^20^ Studies of mild‐to‐moderate depressive symptoms have shown promising outcomes with nicotine patches and chewing gums.^21^ The correlation between nicotine exposure and novel coronavirus pneumonia infection has also been investigated, with the results indicating that prior exposure to nicotine suppresses viral expression, neuroinflammation, and neuronal death.^22^ Research has also found that nicotine reduces the onset of Parkinson's disease, which is mainly related to its ability to reduce microglia activation and its protective effect on dopaminergic neurons.^23^ However, the impact of nicotine on the mouse hippocampal nervous system after CS exposure needs further exploration.

In this study, we investigated the occurrence of hippocampal neuronal inflammation in mice after CS exposure and the effect of nicotine on neuronal inflammation in mice exposed to CS. We found that CS exposure led to the activation of astrocytes via NF‐κB signaling and elevated the expression of Lcn2. These factors collectively contributed to neuronal death, ultimately resulting in the manifestation of depression‐like behavior. Interestingly, nicotine weakened the upregulation of NF‐κB in astrocytes, decreased the expression of Lcn2, and relieved the neuroinflammation, thereby somewhat protecting neurons and reducing depressive symptoms. These findings have important implications for the development of new therapies for depression and cognitive impairment associated with chronic exposure to CS.

## 2 MATERIALS AND METHODS

### 2.1 Animals

Male C57BL/6 mice were procured from Henan Skbex Biotechnology Co., Ltd. (license number SCXK (Yu) 2020–0005) and were housed individually in standard cages and maintained under suitable environmental conditions, including a temperature of 21°C ± 1°C, humidity of 53% ± 3%, and a 12/12‐h light/dark cycle. Standard food and water were provided ad libitum. All experimental procedures were conducted with the approval of the Animal Care and Use Committee of the Anhui University of Science and Technology and in strict compliance with the guidelines set forth by the National Institutes of Health for the Protection and Use of Laboratory Animals (NIH Publication No. 8023, revised 1978).

### 2.2 The CS exposure and nicotine administration

In the initial experiment, 24 mice were randomly and equally allocated into two groups: the Sil group was exposed to CS at a concentration of 20 mg/mL in a volume of 50 μL, while the Veh group was given an equal volume of saline. Over 2 weeks, CS or saline was administered via nasal drip every 3 days for five times in total, with the experiment running for a total of 3 weeks.

For the subsequent experiments, the mice were randomly allocated into four groups with 12 mice per group: (1) Veh, the control group, which received water containing 1% saccharin and 50 μL of saline via nasal drip; (2) Nic, which was administered 200 μg/mL nicotine in 1% saccharin and 50 μL of saline via nasal drip; (3) Sil, which was administered CS at a concentration of 20 mg/mL in a volume of 50 μL and received water containing 1% saccharin; and (4) Nic + Sil, which received nicotine combined with CS exposure. The initial concentration of nicotine was 50 μg/mL for the first 2 days and was then increased by 50 μg/mL every 2 days until it reached 200 μg/mL. The nicotine containers were enveloped in aluminum foil and shielded from light and the nicotine was consistently administered until the completion of the experiment. The nicotine solution was refreshed every other day, and saline or CS was administered via nasal drip every 3 days for five times in total. Behavioral experiments were performed and tissue samples were collected at weeks 3, 6, and 9. The CS and nicotine were obtained from Sigma‐Aldrich (Shanghai) Trading Co. Ltd. Briefly, 80% of the CS particles have a diameter ranging from 1 to 5 μm, and the particles were suspended in saline and sonicated for 10 min before use.

### 2.3 Behavioral tests

Behavioral tests were conducted at weeks 3, 6, and 9, with mice being pre‐adapted to the experimental environment for a period of 3 days. These tests comprised the open‐field test (OFT), elevated plus maze (EPM), and marble burying test (MBT), which were conducted sequentially with a 1‐h interval between each experiment.

During the OFT, each mouse was placed in the center of a transparent box measuring 30 × 30 × 35 cm^3^ and allowed to explore freely for 30 min. This experiment was conducted from 13:00 to 17:00, and all boxes were thoroughly cleaned with 70% alcohol between each test. The depression‐like moods of the mice were assessed based on their total distance traveled (cm) and percentage of immobility.

The EMP comprised two open arms measuring 30 × 5 cm^2^, two closed arms of the same size and a central platform measuring 5 × 5 cm^2^. The entire apparatus was elevated 50 cm above the ground, and the mice were placed in the center facing an open arm and allowed to explore freely in dim light for 5 min. A thorough cleaning with 70% alcohol was conducted after each test to ensure the accuracy of subsequent measurements. Depression‐like behaviors were assessed based on total distance traveled (cm) and frequency of open‐arm entries. EthoVision XT software was used for data collection and analysis in both the EPM and the OFT.

During the MBT, each mouse was placed in a 28 × 18 × 12 cm^3^ acrylic cage filled 5 cm deep with uniformly padding, clean glass marbles measuring 1.5 cm in diameter and was allowed to explore for 30 min. After each test, the number of marbles buried in the corn cob pellets was counted; glass spheres with an embedded volume greater than two‐thirds were recorded.

### 2.4 Determination of Evan's blue content in brain tissue

A 2 mL/kg body weight injection of a 5% Evan's blue (EB) solution was administered into the retro‐orbital vein of mice at an approximate angle of 30° to the face. One hour post‐injection, the mice were humanely euthanized via intracardiac perfusion and cervical dislocation, and brain tissues were promptly extracted on ice. The tissues were then weighed, placed in 1.5‐mL Eppendorf tubes, and incubated with 500 μL of formamide at 55°C for 24–48 h. After centrifugation at 1500*g* for 10 min, the absorbance of the 610‐nm band was measured and the EB content (ng/mg) was calculated.^24^

### 2.5 Histologic staining

Apical perfusion was conducted in each group of mice using 0.1 M PBS and 4% paraformaldehyde (PFA), followed by removal and storage of a coronal section containing the bilateral hippocampus in 4% PFA at 4°C for 72 h. The slice thickness was 4 mm, extending from bregma −0.94 to −3.88 mm. Subsequently, the brains were dehydrated using 20% and 30% sucrose solutions before being embedded in optimal cutting temperature (OCT) compound and rapidly frozen for 10 min. Conversely, the brains intended for paraffin sectioning were immersed in alcohol solutions (75%, 85%, 95%, and 100%) and subsequently paraffin‐embedded.

For Nissl staining, 5‐μm‐thick paraffin‐embedded brain sections were stained with 1% thionin. The cells exhibiting neuronal body atrophy and intense cytoplasmic staining are classified as damaged neurons.^25^

A 5‐mm hippocampal section was dewaxed in xylene for 30 min and dehydrated in gradient alcohol for 30 min. A 0.01 mol/L sodium citrate buffer was applied to retrieve antigens, and the tissue was subsequently blocked with 5% bovine serum albumin (BSA) for 1 h at room temperature. The primary antibody was incubated overnight at 4°C. After being washed with 0.1 M PBS, the sections were incubated with goat anti‐rabbit IgG (H&L) secondary antibody for 40 min at room temperature. Staining was performed with 3,3‐diaminobenzidine tetrahydrochloride (DAB) and hematoxylin, and images were captured using a VS‐200 scanning microscope (Olympus).

The One‐step TUNEL Apoptosis Assay Kit (Beyotime, C1086) was used to detect apoptotic cells. FITC‐labeled dUTP was added to confirm the presence of apoptotic cells, which appeared as green fluorescence under the microscope. ImageJ software was used to calculate the total number of cells and apoptotic cells.

For immunofluorescence staining, before being blocked with 5% of BSA for 1 h at room temperature, the samples underwent antigen repair for 15 min. Then, 30‐μm‐thick tissue samples were incubated with primary antibodies at 4°C overnight. After being washed with 0.1 M PBS, the sections were incubated with secondary antibody for 1 h at room temperature and cell nuclei were stained with 4,6‐diamidino‐2‐phenylindole (DAPI). Finally, the stained sections were sealed using an anti‐fluorescence quenching agent. A comprehensive list of primary and secondary antibodies can be found in Table 1.

**TABLE 1:** Primary antibodies used in immuno‐staining.

Antigen	Host	Dilution ratio	Company
NeuN	Rabbit	1:300	CST,12943S
IL‐6	Rabbit	1:300	Bioss, bs‐0782R
CD31	Mouse	1:1000	CST, 3528S
Cldn‐5	Mouse	1:50	Santa cruz, sc‐374,221
Albumin	Rabbit	1:300	Bioss, bs‐2256R
TUNEL			Beyotime, C1088
Iba1	Rabbit	1:300	Wako,019–19,741
Lcn2	Rabbit	1:300	Bioss, bs‐1373R
GAFP	Rabbit	1:300	Abcam, ab7260
GFAP	Mouse	1:200	CST, 3670S
Lcn2	Goat	1:200	R&D, AF‐1857
Iba1	Goat	1:200	Novus, NB100‐1028
GAPDH	Rabbit	1:10000	Proteintech, 10,494‐1‐AP
Bax	Rabbit	1:200	Proteintech 50,599‐2‐IG
DAPI		1:1000	Beyotime, C1002
Goatanti‐rabbitIgGH&L	Donkey	1:400	Abcam, ab6721
Goat anti‐Rabbit IgG H&L Alexa Fluor 488	Goat	1:400	Life Technology, A‐11006
Goat anti‐mouse IgG H&L Alexa Fluor 594	Goat	1:400	Life Technology, A‐11037
Donkey anti‐rabbit Alexa 594	Donkey	1:400	Abcam, ab150076
Donkey anti‐goat Alexa 488	Donkey	1:400	Abcam, ab150129
Donkey anti‐mouse Alexa fluor 647	Donkey	1:400	Invitrogen, A‐31571
NF‐κB	Rabbit	1:600 (WB)	Bioss, bs‐2321R
HRP‐conjugated Affinipure Goat Anti‐Rabbit IgG (H + L)	Goat	1:1000 (WB)	Proteintech, SA00001‐2

Glial cells were visualized and captured with confocal laser scanning microscopy (Olympus FV‐3000) and subsequently analyzed using cellSens software (Olympus). The scan thickness was 9 μm and the scan step size was 0.3 μm. Z‐series through the cells were used to create 3D objects from the polygon stacks (1024 × 1024 pixels) obtained using a 40× objective lens.

### 2.6 Sholl analysis

For Sholl analysis, changes in synapse length and number were monitored by quantifying the number of cellular synapses intersecting concentric circles of 1‐μm radii. The experimental protocol of Tavares et al. was followed, with concentric circles spaced at intervals of 1 μm.^26^ Three mice per group, with 6–8 cells per mouse, were selected for analysis. This method allows for a precise assessment of synaptic morphology and plasticity. Image analysis and processing were performed using the “Concentric Circles” plug‐in of Fiji‐ImageJ software.^27^ Quantification of data pairs and determination of the number and length of astrocyte processes were performed via ImageJ analysis.

### 2.7 Enzyme‐linked immunosorbent assay

Blood was collected from the right eye socket, transferred to a heparin‐containing tube, thoroughly mixed and centrifuged at 3000 rpm for 15 min. The supernatant was then collected for measurement of the levels of interleukin‐6 (IL‐6), IL‐1β, and TNF‐α using Mouse IL‐6 ELISA Kit (ABclonal, RK00008, China), Mouse IL‐1 beta ELISA Kit (ABclonal, RK00006) and Mouse TNF‐alpha ELISA Kit (ABclonal, RK00027), respectively. Briefly, specific standards and sera for each kit were added to a pre‐coated 96‐well plate and reacted with the primary antibody. Subsequently, the sample was incubated with secondary antibody conjugated to HRP. After the unbound antibody was washed away, the reaction was then conducted using 3,3′,5,5′‐tetramethylbenzidine (TMB). The absorbance of the specimens at 450 nm was measured using an Epoch2 (BioTek, USA) full‐wavelength microplate spectrophotometer.

### 2.8 RNA sequencing and quantitative reverse transcription polymerase chain reaction analysis

During tissue preparation, anesthetized mice were transcardially perfused with 0.1 M PBS (pH 7.4) at 4°C. RNAlater reagents (Sigma) were then used for RNA sequencing and quantitative polymerase chain reaction analysis (qPCR) analysis. The hippocampus was extracted from mice in the Veh, Sil, Nic + Sil, and Nic groups for RNA sequencing, followed by cDNA library construction using NEBNext®. Quality control was performed using Illumina Casava version 1.8 base identification and Phred scores. Alignment with reference sequences from the Ensembl website (http://www.ensembl.org) was conducted using HISAT2 (v2.2.1), and FPKM was counted for each gene using HTSeq (v0.13.5). Differentially expressed genes (DEGs) were analyzed using the R package deseq2 (1.30.0), with DEGs identified using fold‐change cut‐off values greater than 1 and *p*‐values less than 0.05. Pathway enrichment analysis was performed using Gene Ontology (GO) analysis.

For qPCR analysis, total RNA was extracted from the hippocampus using TRIzol (Thermo Fisher Scientific), followed by cDNA preparation using ABScript III RT for qPCR (RK20408; ABclonal). qPCR analyses were then performed using 2× SYBR Green Fast qPCR Mix (RK 21204; ABclonal). A QuantStudio 3 qPCR System (Thermo Fisher Scientific) was used for qPCR, and relative expression was calculated using the 2^−ΔΔct^ method with QuantStudio™ Design & Analysis Software (v1.3.1). Table 2 lists the primers, which were purchased from Sangon Biotech.

**TABLE 2:** The primer sequences used for real‐time quantitative polymerase chain reaction analyses.

GENE	Forward (5′‐ 3′)	Reverse (3′‐ 5′)
Lcn2	ACCACGGACTACAACCAGTT	ACACTCACCACCCATTCAGT
S100a8	TCGTGACAATGCCGTCTGAACTG	TCTTGTAGAGGGCATGGTGATTTCC
S100a9	GGAAGCACAGTTGGCAACCTTTATG	TGTGTCCAGGTCCTCCATGATGTC
Ly6a	TCCCATTTGAGACTTCTTGCCCATC	CCACAATAACTGCTGCCTCCTGAG
Chil3	GCCCACCAGGAAAGTACACAGATG	GACCTCAGTGGCTCCTTCATTCAG
FAS	GGAGGCGGGTTCGTGAAACTG	AACGGGCTGAATTTCTGATGGTCTC
GAPDH	GTGGGTGCAGCGAACTTTAT	CACTGAGCATCTCCCTCACA

### 2.9 Western blot

Thirty micrograms of hippocampal proteins from mice were separated using 10% sodium dodecyl sulfate‐polyacrylamide gel electrophoresis (SDS‐PAGE) (Beyotime Biotechnology) and then transferred to a polyvinylidene fluoride membrane (PVDF) (Millipore). The membrane was washed thrice with Tween 20 (TBST) Tris‐salt solution and blocked with 5% BSA or skim milk for 1 h. The primary antibody, diluted with 5% BSA‐TBST, was then applied and incubated overnight at 4°C. Following a rinse with TBST, the membrane was incubated with HRP‐conjugated secondary antibody (Proteintech) at room temperature for 1 h. The film was exposed to enhanced chemiluminescence (ECL) (Millipore) for 30 s before evaluation using an Amersham ImageQuant 800 (Cytiva) and subsequent quantification using ImageJ software. The study used three groups of mice for experimentation, and Table 1 lists the primary antibodies.

### 2.10 Statistics analysis

Data are presented as mean ± standard deviation (SD). The statistical analyses included one‐way ANOVA and an independent samples t‐test. Weight loss was analyzed using two‐way ANOVA with Bonferroni's multiple comparisons. Prism 7.0 software was used to perform the statistical analyses, where *p* < 0.05 was set as the level of significance for determining any significant differences. Shapiro–Wilk normality test was used to determine the normality of the data.

## 3 RESULTS

### 3.1 CS exposure damages the BBB

**FIGURE 1:**
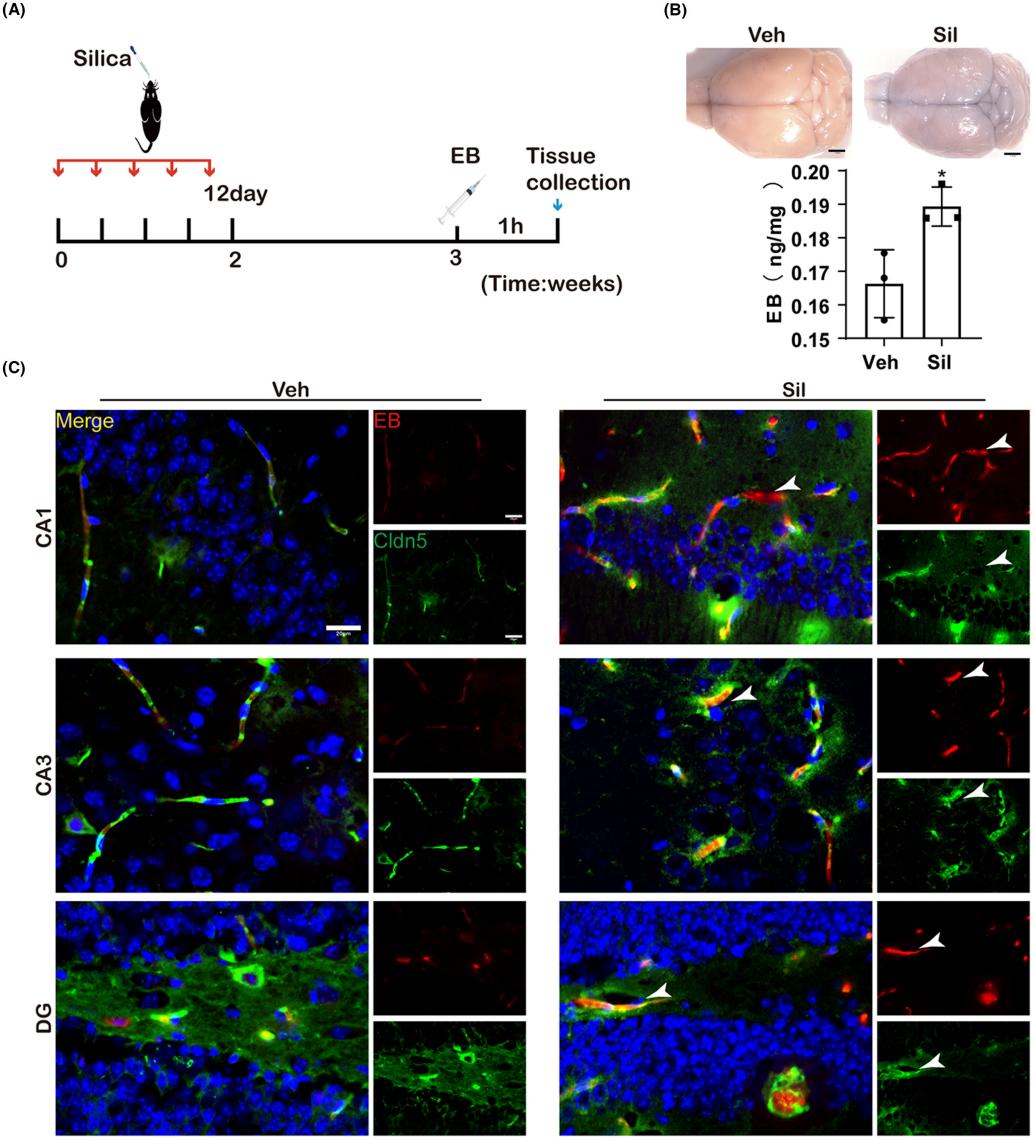
CS exposure increased the damage of the BBB in mice. (A) The experimental design of the destructive effect of CS exposure on the blood–brain barrier. (B) Photographs of EB dye extravasation into the brain and EB content in the hippocampus. Scale bar: 1.5 mm. *p < 0.05. (C) Representative graph showing the BBB leakage. Immunofluorescent staining for Cldn‐5 (green) and EB (red) in CA1, CA3, and DG areas. White Arrow Cldn5 Decrease and EB Exudation. Scale bar: 20 μm. *n* = 3 per experimental group.

To investigate the effects of CS exposure on BBB integrity, we used two groups of mice (Sil and Veh). The experimental procedure is outlined in Figure 1A. EB, an azo dye known for its affinity for plasma albumin, was used to indicate BBB integrity. After EB injection and rapid brain removal, formamide was used to extract the EB content in the hippocampus. CS exposure caused a significant increase in EB content in the hippocampus compared to the control group (*p* = 0.0269), as shown in Figure 1B. Additionally, EB colocalized with FITC‐conjugated claudin‐5 in the hippocampus of Veh group mice at week 3 post‐exposure, thereby verifying the BBB integrity. CS exposure was found to increase BBB permeability, resulting in albumin leakage from the peripheral vasculature into the brain, as evidenced by increasing co‐staining of albumin with EB (Additional file 1: Figure S1C). Moreover, we found a decrease in CD31 expression and a significant increase in IL‐6 expression in the hippocampal CA1 region of CS‐exposed mice (Additional file 1: Figure S1A,B). Taken together, these results suggest that CS exposure can disrupt the BBB.

### 3.2 CS exposure promotes astrocyte activation and increases Lcn2 expression

**FIGURE 2:**
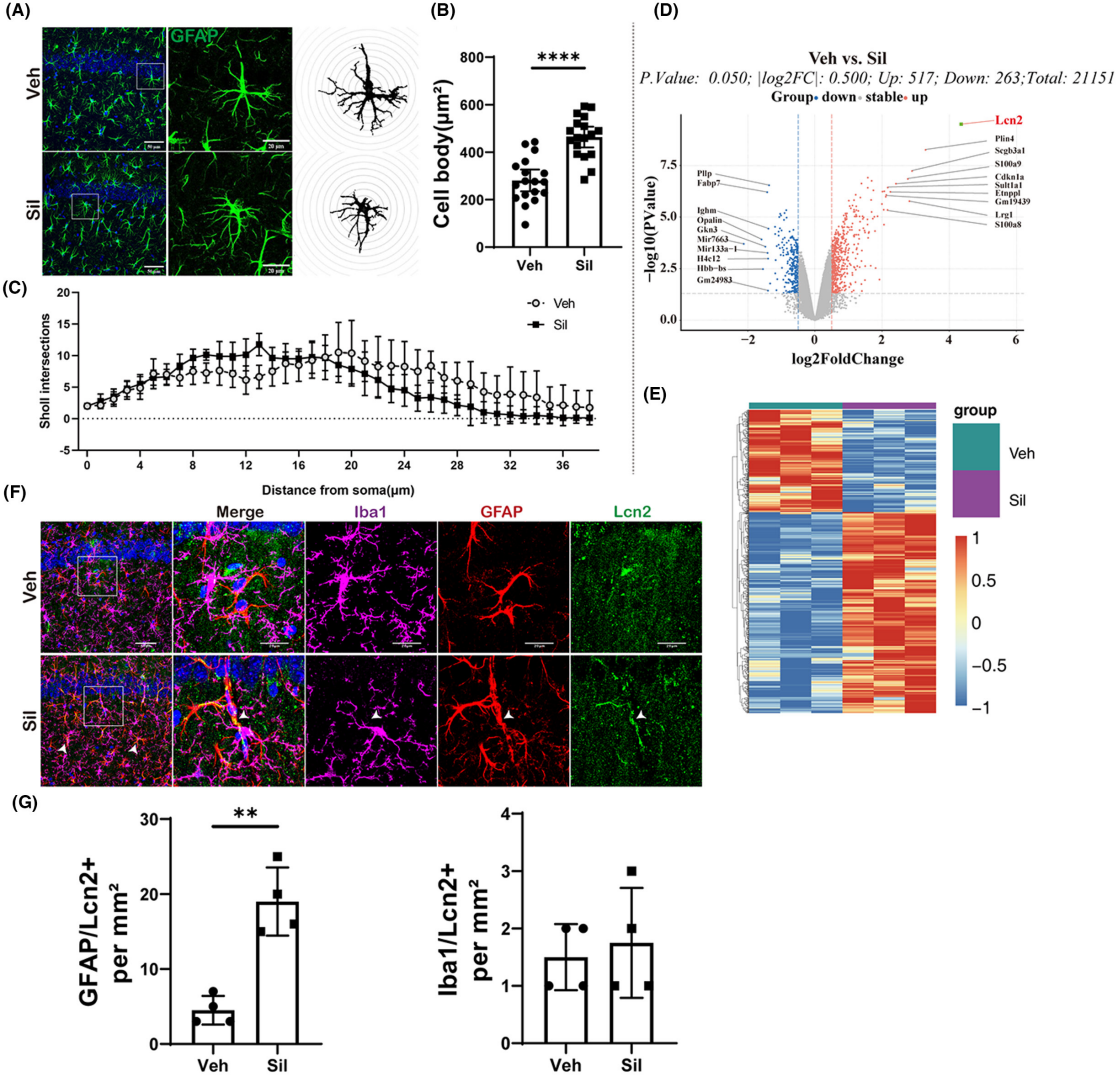
*CS exposure* enhanced hippocampal CA1 astrocyte activation and increased LCN2 expression in these cells. (A) Representative images of immunofluorescence‐stained astrocytes (GFAP). Scale bar: 50 μm and 20 μm. (B) Analysis of astrocyte cell soma, *p* < 0.0001. (C) Sholl analysis of synaptic length changes in astrocytes, *n* = 4 per experimental group. (D, E) Volcano maps and heat maps presenting differential genes (DEGs) in hippocampal regions after CS exposure. Fold change = 1.2, *p* < 0.05, *n* = 3 per experimental group. (F) Representative pictures of GFAP, Iba1, Lcn2 immunofluorescence staining. The white arrow indicates the cells with co‐expression of GFAP and Lcn2. Scale bar: 50 μm and 20 μm. (G) Statistical results for GFAP+/Lcn2+ cells and Iba1+/Lcn2+ cells. *n* = 4 per experimental group, **p < 0.05.

In the Sil group, the astrocytic cell soma exhibited significant swelling (*p* < 0.0001) (Figure 2A,B). In addition, there was a substantial increase in synapses within the 9–13‐μm range (*p* < 0.05) but a drastic reduction in synapses longer than 20 μm (*p* < 0.05), as well as overproduction of glial fibrillary acidic protein (GFAP) (Figure 2C), suggesting astrocyte activation following CS treatment.

Furthermore, we sequenced the mouse hippocampal transcriptome, identifying differential expression of 517 upregulated and 263 downregulated genes in the Sil group compared to the Veh group, with Lcn2 the most highly upregulated gene (log2 (fold‐change) = 4.35) (Figure 2D,E), as indicated on volcano plot and heat maps. Thus, CS exposure influenced the activation of hippocampal glial cells in mice. Immunofluorescence staining revealed that glial cell activation in the CA1 region of the hippocampus of Sil group mice was marked compared to the Veh group, with increased astrocyte numbers. Moreover, there was a notable upregulation of Lcn2 expression, primarily in astrocytes (*p* < 0.01) (Figure 2F,G), as indicated by the white arrows in the figure. Lcn2 expression was similar in the CA3 and DG regions and in the CA1 region. Exposure to CS induced damage or death of hippocampal neurons and increased Lcn2^+^ reactive astrocytes in the CA3 and DG regions of the hippocampus. Nicotine had a neuroprotective effect, reducing neuronal injury or death and relieving anxiety and depression (data not shown).

### 3.3 Nicotine reduces CS exposure‐induced astrocyte activation by weakening NF‐κB signaling

**FIGURE 3:**
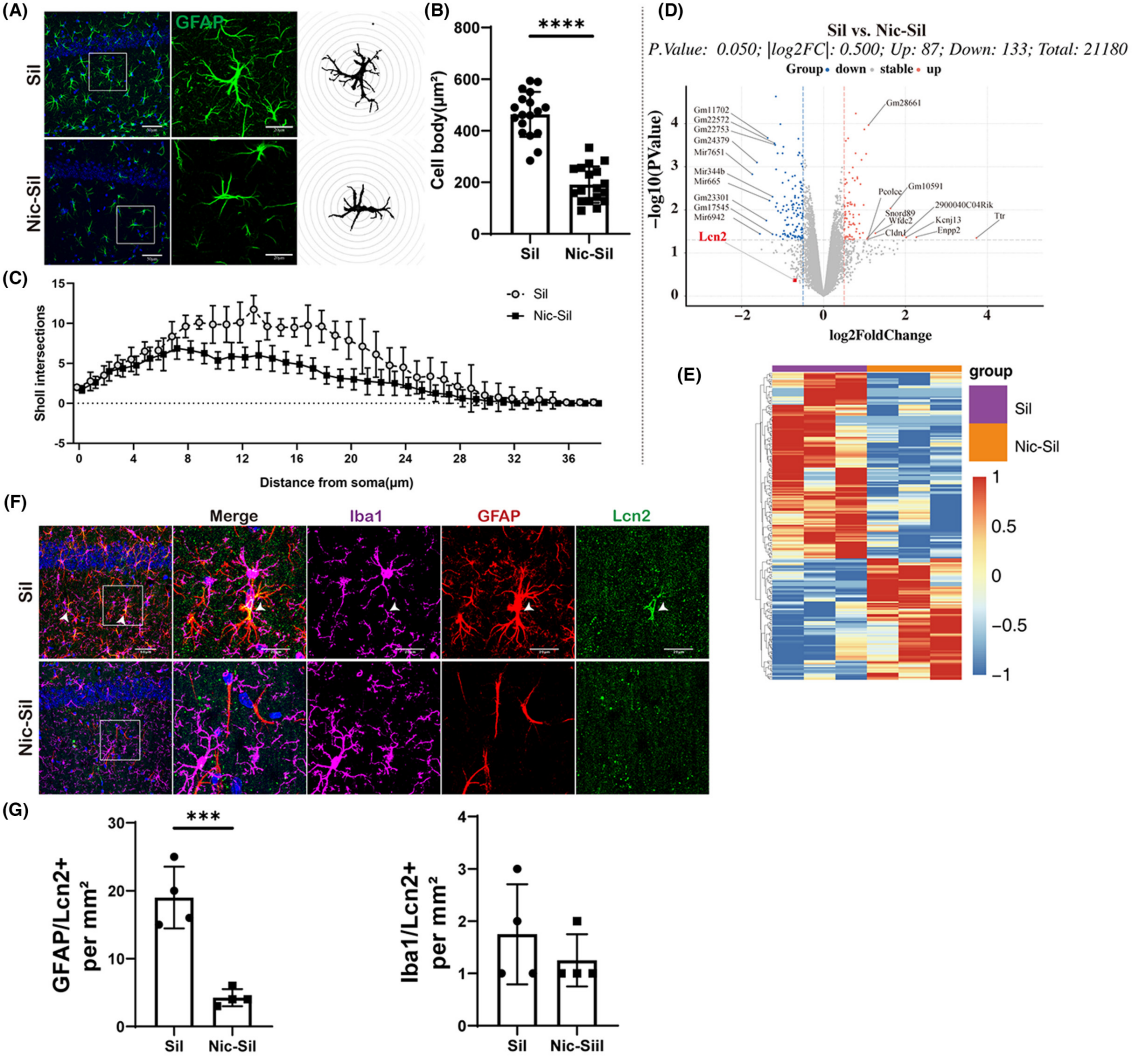
Nicotine inhibited CS exposure‐induced astrocyte activation and Lcn2 production. (A) Representative images of immunofluorescence‐stained astrocytes (GFAP). Scale bar: 50 μm and 20 μm. (B) Analysis of astrocyte cell soma, *p* < 0.0001. (C) Sholl analysis of synaptic length changes in astrocytes, *n* = 4 per experimental group. (D, E) Volcano maps and heat maps presenting differential genes (DEGs) in hippocampal regions after CS exposure. Fold change = 1.2, *p* < 0.05, *n* = 4 per experimental group. (F) Representative pictures of GFAP, Iba1, Lcn2 immunofluorescence staining. The white arrow indicates the cells with co‐expression of GFAP and Lcn2. Scale bar: 50 μm and 20 μm. (G) Statistical results for GFAP+/Lcn2+ cells and Iba1+/Lcn2+ cells, *n* = 4 per experimental group, **p < 0.05.

To further explore the impact of nicotine on the nervous system, we initially investigated the effects of nicotine on astrocytes. Our findings revealed that, compared to the astrocytes in the Sil group, the astrocytes in the Nic‐Sil group displayed a reduced cell body size (*p* < 0.00001) and decreased synaptic density, indicating a resting state (Figure 3A–C). Analysis of the volcano plot and heat map of DEGs corroborated our findings, with a total of 21,180 DEGs in the astrocytes of the Nic‐Sil versus. Sil groups, comprising 87 upregulated genes and 133 downregulated genes, including Lcn2 (Figure 3D,E). These results suggested that nicotine could mitigate astrocyte activation. In addition, immunofluorescence staining demonstrated a reduction in Lcn2 levels produced by astrocytes, with a significant decrease in the number of Lcn2^+^/GFAP^+^ cells (*p* < 0.0001) (Figure 3F,G).

**FIGURE 4:**
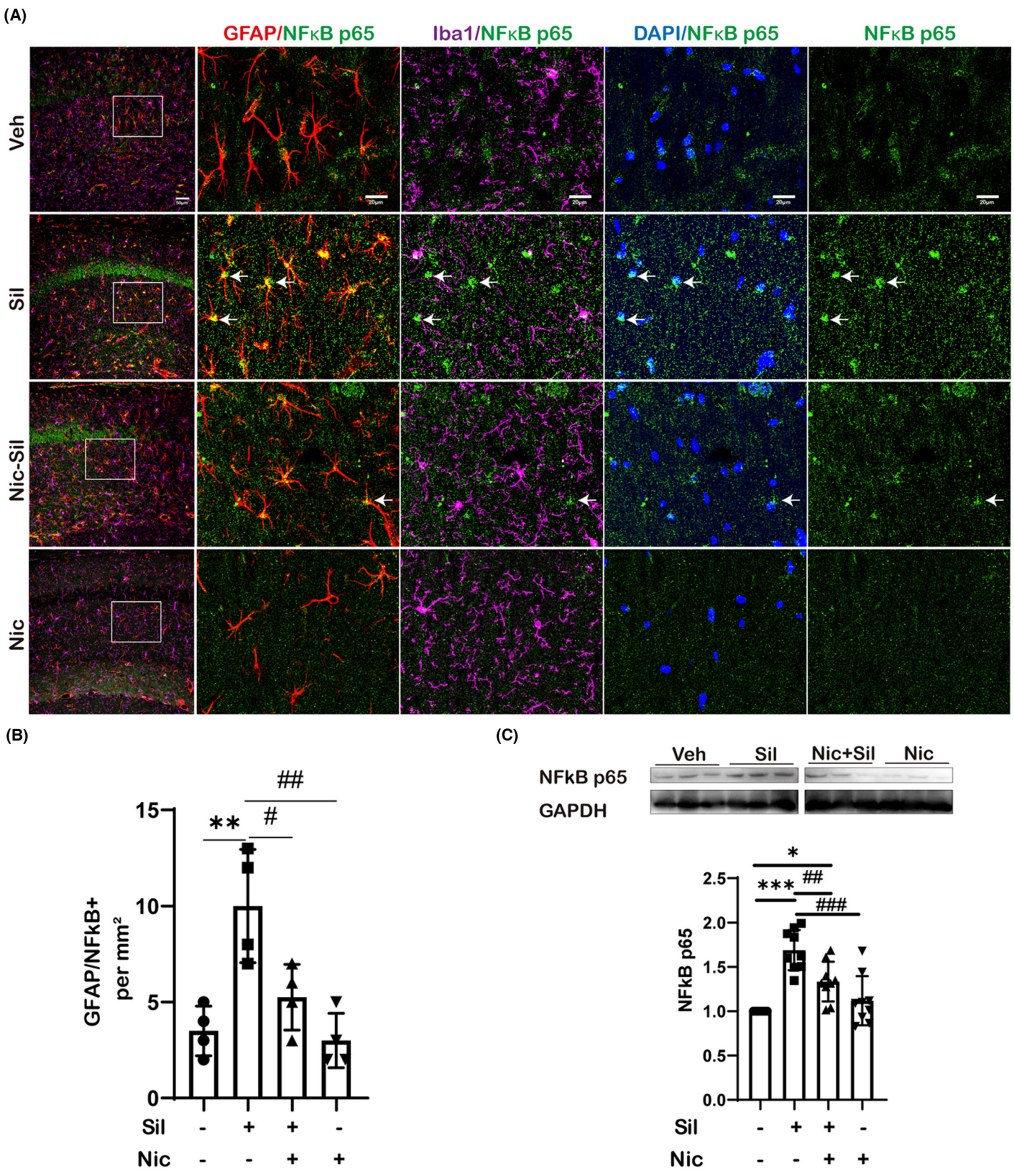
Nicotine suppressed the NF‐κB in astrocytes, leading to a reduction in astroglial activation. (A) Representative pictures of GFAP, Iba1, NF‐κB immunofluorescence staining. White arrows indicate NF‐κB expression in astrocytes. NF‐κB entry into the nucleus was found to be evident in the Sil group. *n* = 4 per experimental group, Scale bar: 50 μm and 20 μm. (B) The statistical results of GFAP+/NF‐κB+ cells (**p < 0.01 vs Veh; ^#^ *p* < 0.05; ^##^ *p* < 0.01 vs Sil). (C) The NF‐κB protein expression and quantification, *n* = 4 per experimental group, *p < 0.05; ***p < 0.001 vs Veh; ^##^ *p* < 0.01; ^###^ *p* < 0.001 vs Sil.

Through further exploration of the pathways involved in the activation of astrocytes, we discovered that exposure to CS resulted in a significant upregulation of NF‐κB expression in astrocytes and a notable increase in the quantity of GFAP^+^/NF‐κB^+^ cells (*p* < 0.01). In contrast, nicotine substantially decreased astrocytic NF‐κB expression, as evidenced by our findings in Figure 4A,B. Additionally, quantitative protein analysis indicated that CS exposure upregulated NF‐κB expression while nicotine attenuated it (Figure 4C). These findings provided valuable insights into astrocytic activation mechanisms and may yield promising targets for the development of treatment strategies for related neurological disorders.

### 3.4 CS exposure results in the expression of pro‐inflammatory and pro‐apoptotic genes, whereas nicotine has an inhibiting effect

**FIGURE 5:**
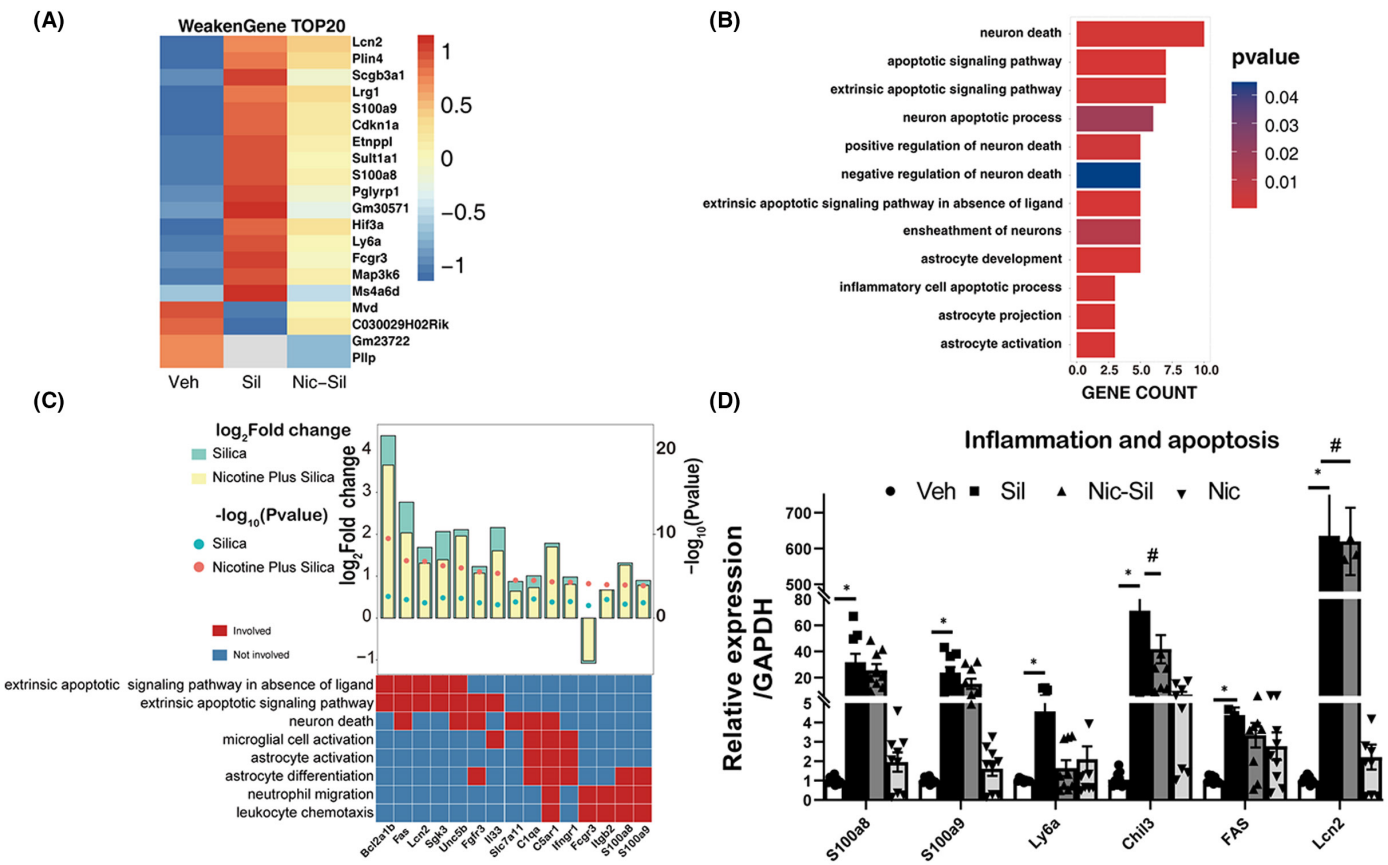
Nicotine reduced pro‐inflammatory and pro‐apoptotic gene expression. (A) Heat map of the top 20 Weaken Genes screened by transcriptome sequencing, *p* < 0.05, Fold change >1.2, *n* = 3 per experimental group. (B, C) GO analysis of Weaken Genes and search for pathways associated with neurological inflammation or neuronal cell death. (D) Validation of gene expression associated with inflammation, and apoptosis using qPCR, *n* = 4 per experimental group, *p < 0.05 vs Veh group; ^#^ *p* < 0.05 vs Sil group.

The inflammation‐related pathways from the GSEA database were downloaded. We identified 227 weakened genes and have presented the top 20 genes in a heat map. These weakening genes were characterized by their significant up‐ (or down‐) regulation in the Sil group, but not in the Nic + Sil group (Figure 5A,B). Among them, Lcn2, involved in apoptosis and inflammatory processes, stood out as the most drastically altered protein. In situ immunofluorescence staining showed that astrocytes are the primary source of Lcn2 production. The expression of Lcn2 was significantly elevated in the Sil group compared to the Veh group, while no changes were observed in the Nic group. Notably, Lcn2 expression was reduced in the Nic + Sil group relative to the Sil group (Figure 5C). Additionally, GO analysis revealed subtle changes in their expression with respect to cellular components, biological processes and molecular function. The results showed that the DEGs were enriched in neuroinflammation and neuronal death. Specifically, qPCR analysis confirmed that the expression of FAS, Lcn2, S100a8, S100a9, Ly6a, and Chil3 increased significantly after exposure to CS. The genes S100A8/A9, Chil3, Ly6a, and Lcn2 are known to be closely associated with inflammation while FAS is closely associated with neuronal death. This was further corroborated by the observation that CS‐induced injury led to upregulated levels of apoptosis‐related proteins and promoted the expression of inflammatory and apoptotic genes in the hippocampal nervous system (Figure 5D), suggesting that neuronal death occurs as a consequence of CS exposure. However, the protective effect of nicotine was demonstrated in this context because it inhibited the expression of these genes in the hippocampus, thereby safeguarding the viability of the neuronal cells.

### 3.5 CS exposure causes neuronal death in the CA1 region while nicotine is protective

**FIGURE 6:**
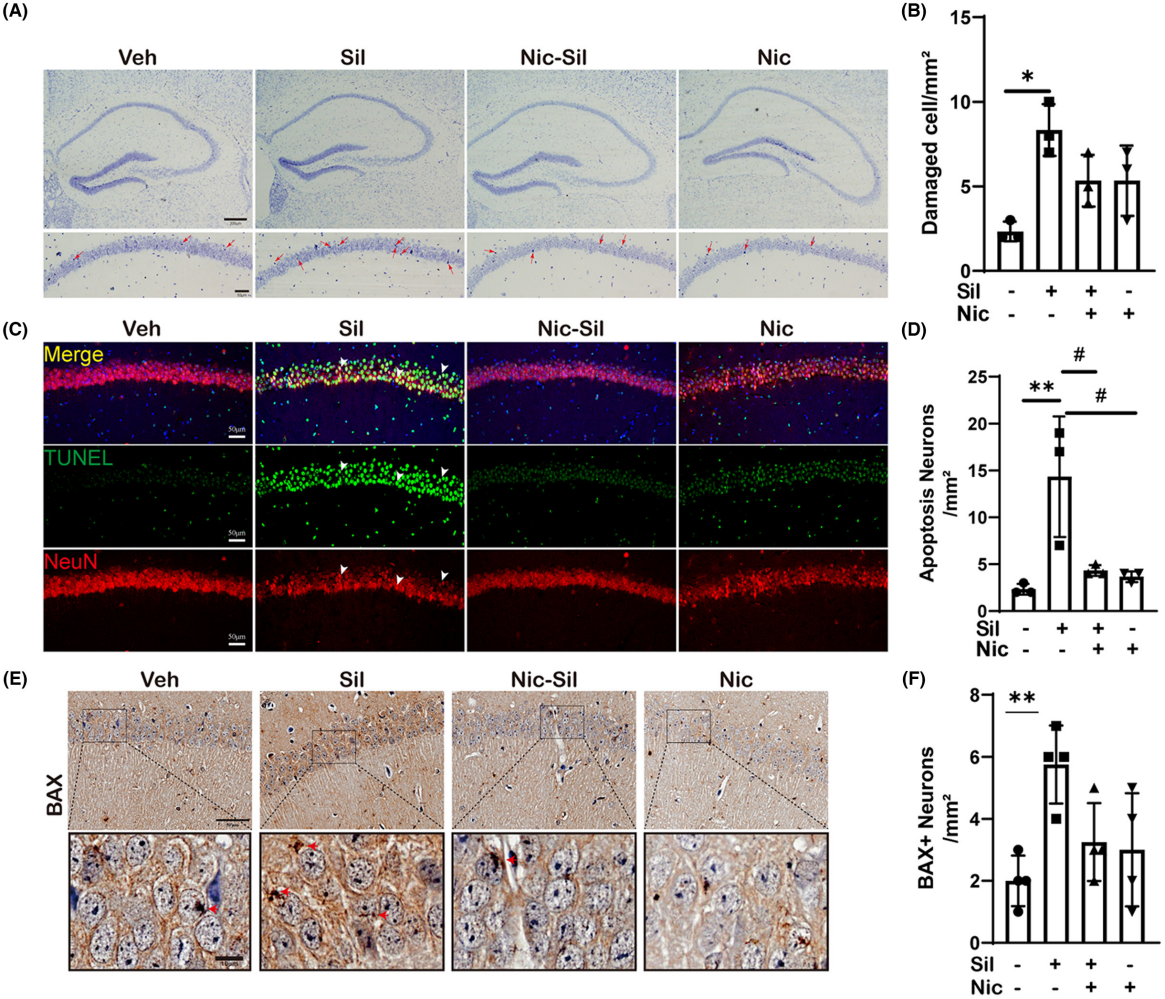
Nicotine reduced neuronal death caused by CS exposure. (A) Representative images of Nissl staining, scale bars: 200 μm and 50 μm. (B) Statistical results on the number of injured neurons, *p < 0.05. (C) TUNEL, NeuN representative immunofluorescence staining results, scale bar: 50 μm. (D) Statistics on apoptotic neuron cells, *n* = 3 per experimental group, *p < 0.05 vs Veh; #*p* < 0.05 vs Sil. (E) Representative images of BAX staining, *n* = 3 per experimental group, scale bars: 50 μm and 10 μm. (F) Statistics on BAX+ neuron cells, *n* = 4 per experimental group, **p < 0.01 vs Veh.

Our observations revealed that the Sil group exhibited markedly greater neuronal damage, particularly in the CA1 region, in ipsilateral Nissl‐stained sections compared to the Veh group. These neurons were significantly characterized by atrophied cytoplasm and damaged nuclei (*p* < 0.05) (Figure 6A,B). Furthermore, there was a substantial increase in the number of TUNEL^+^ cells in the pyramidal layer neurons of the CA1 area within the Sil group when compared to the Veh group (*p* < 0.01) (Figure 6C,D). Similarly, the Sil group exhibited significantly higher levels of BAX^+^ cells compared with the Veh group (Figure 6E,F), indicating an increase in apoptotic neurons. Nissl staining revealed a notable rise in the number of cells with reduced cytoplasm and pyknotic nucleus, while the TUNEL staining results demonstrated a significant increase in apoptotic neurons.

Furthermore, we observed a significant reduction in the number of damaged neurons on the ipsilateral side of the Nissl‐stained slices in the Nic‐Sil group compared to the Sil group, particularly in the CA1 region (Figure 6A,B). In addition, the number of TUNEL^+^ cells in the CA1 pyramidal layer neurons was significantly reduced in the Nic‐Sil group (*p* < 0.05) (Figure 6C,D). Similarly, the number of BAX^+^ cells was significantly reduced in the Nic‐Sil group compared to the Sil group (Figure 6E,F), indicating a decrease in apoptotic neurons. These findings suggested that nicotine had a protective effect on neurons under CS exposure conditions. TUNEL, BAX, and Nissl staining confirmed these findings: nicotine significantly reduced neuronal death and promoted neuronal survival.

The removal of damaged neurons is essential for maintaining and remodeling neuronal networks. In this process, astrocytes and microglia play a significant role. When neurons suffer damage, they release cytokines such as CX3CL1 to activate astrocytes and microglia. To investigate the mechanism of neuronal clearance, we used immunofluorescence staining to observe the “triad” structure. In this structure, activated astrocytes extended their tentacles, physically separating damaged neurons from normal neurons and dividing the cells into smaller clusters. At the same time, the activated microglia engulfed and destroyed the damaged neurons, blocking the spread of the damage and thereby effectively preventing inflammation. By comparing the results of the Veh and Sil groups, we found that the number of triads in the CA1 region was significantly increased in the Sil group. On the other hand, the results were not significantly changed in the Nic + Sil group. In addition, we also obtained similar results in the CA3 and DG regions of the hippocampus (Additional file 2: Figure S2A–C). Regarding the weakened genes correlation heat map, exposure to CS induced a significant upregulation of astrocyte activation, particularly the expression of genes related to pan‐astrocytic markers (S1pr3, Timp1, Timp2, Hif3α, Stat3, Psmb8, and Lcn2) and type I astrocytes (Srgn, Amigo2, Fkbp5, IL17ra, and Slc10a6). In contrast, type II astrocytes (Aqp4, Tgm1, and Ptx3) were significantly downregulated after CS activation. (Additional file 3: Figure S3A). Additionally, the expression levels of complement C3, C1qa, C1qb, C1qc, and Trem2, highly associated with neuronal death, exhibited significant upregulation, while brain‐derived neurotrophic factor (BDNF) expression was significantly decreased (Additional file 3: Figure S3B). On the other hand, nicotine significantly weakened the expression of PAN and A1 markers and promoted the expression of A2 markers and genes related to neuronal survival.

### 3.6 Nicotine attenuates inflammation and abnormal behaviors in mice exposed to CS

**FIGURE 7:**
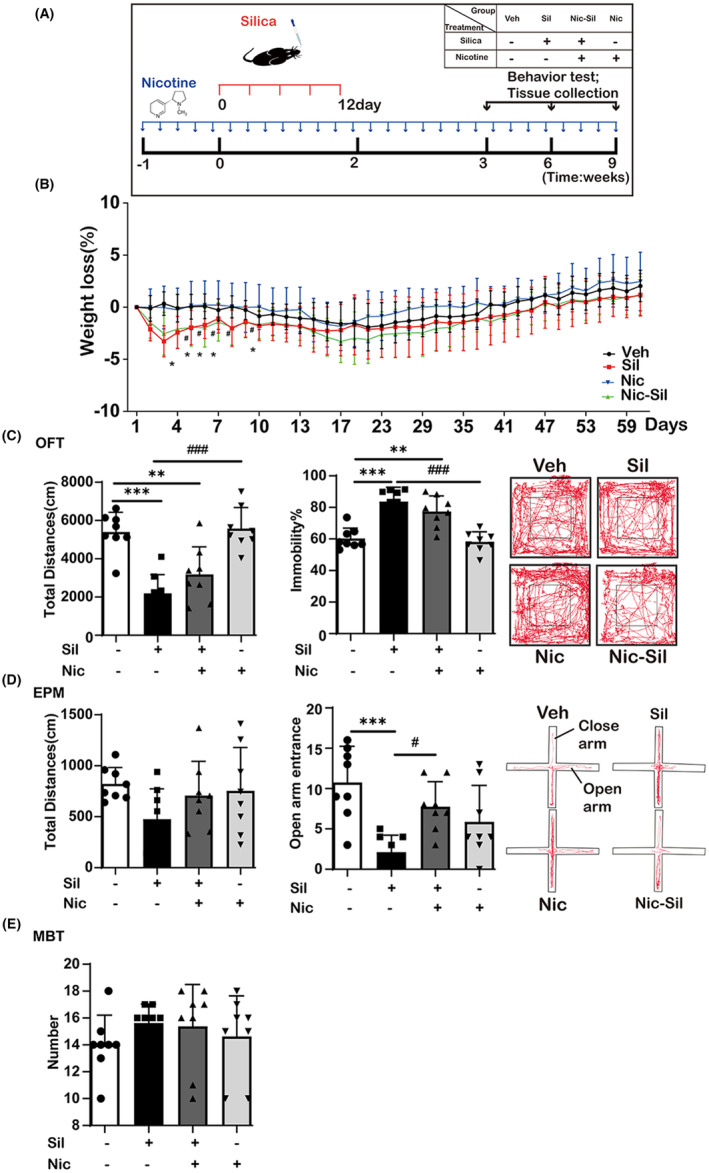
Nicotine alleviated CS‐mediated depression‐like behavior. (A) The experimental design and behavioral testing. The mice were randomly divided into four groups: Veh, Sil, Sil + Nic, and Nic group. After acclimatization, mice were continuously ingested with nicotine (Nic, Nic + Sil) or saccharin (Sil, Veh) sodium. Saline (Nic, Veh) or CS (Nic + Sil, Sil) was then administered via intranasal drip every 3 days for five times in total. Behavioral tests were performed at weeks 3, 6, and 9; organs were collected after testing. (B) Weight loss (%) was measured every 2 days. Weight loss = (Daily weight—initial weight) / initial weight, *p < 0.05 Sil vs Veh group; ^#^ *p* < 0.05 Nic + Sil vs Veh group. (C) The total distances and immobility time (%) in the OFT at 3 weeks, **p < 0.01; ***p < 0.001 vs Veh group; ^###^ *p* < 0.001 vs Sil group. (D) The total distances and open arm entrance in the EPM at 3 weeks, ***p < 0.001 vs Veh group; ^#^ *p* < 0.05 vs Sil group. *n* = 12 per experimental group. (E) The number of beads buried in the MBT at 3 weeks, *p < 0.05 vs Veh group.

To examine the impact of CS exposure on mouse behavior and the inflammatory response and to explore the potential involvement of nicotine in this process, mice were randomly divided into four groups: Veh, Sil, Nic + Sil, and Nic. Behavioral testing was conducted and hippocampal samples were collected at weeks 3, 6, and 9 post‐exposure (Figure 7A). Notably, the Sil or Nic + Sil groups exhibited a significant reduction in body weight from day 3 to day 8 post‐exposure compared to the Veh group (*p* < 0.05) (Figure 7B)

Depression‐like behavior in mice was evaluated by implementing three behavioral tests: OFT, EPM, and MBT. The OFT was used to gauge mood changes by measuring the total distance traveled and immobility time. Results from week 3 showed that, compared to the Veh group, both the Sil (*p* < 0.0001) and Nic + Sil (*p* = 0.003) groups had a significantly lower total distance and increased immobility. Conversely, the Nic + Sil group exhibited an increasing trend in total distance and a decrease in immobility compared to the Sil group (Figure 7C).

In the context of the EPM, at 3 weeks of treatment, the Sil group displayed a significant reduction in the frequency of mouse entry into the open arms compared to the Veh group (*p* = 0.0004), indicating a diminishing trend in exploratory behavior. Notably, the Nic + Sil group did not exhibit any significant variance compared to the Veh group. Additionally, the results revealed that the Nic + Sil group showed an increase in the frequency of entry into the open arm and in total locomotion compared to the Sil group (Figure 7D). Unlike in previous studies, exposure to CS or nicotine consumption did not increase the number of buried marbles (Figure 7E). ELISA analysis revealed significant upregulation of IL‐6 expression in the blood. In contrast, IL‐β and TNF‐α levels were non‐significantly elevated (Additional file 4: Figure S4A–C). Collectively, our findings suggest a potential association between behavioral alterations induced by CS exposure and increased blood inflammatory factors. Notably, mice exposed to CS showed persistent depression‐like behavior, as evidenced by behavioral assays conducted at weeks 6 and 9. However, the nicotine‐induced effect progressively weakened and was nearly absent by week 9 (Additional file 5: Figure S5A–F).

Taken together, these data suggest that exposure to CS leads to the development of depression‐like behaviors. However, nicotine attenuates CS‐induced depression‐like behaviors.

## 4 DISCUSSION

Exposure to particulate matter can contribute to the development of depression.^28^ Furthermore, workers in such industries often have smoking habits, which may be attributed to the ability of nicotine to alleviate depressive symptoms.^29^ The aim of the present study was to examine the impact and underlying mechanisms of CS exposure on neuroinflammation and neuronal apoptosis, along with the potential role of nicotine in alleviating neuroinflammation and enhancing neuronal resilience. The primary discovery of this investigation was that exposure to CS resulted in BBB impairment, allowing inflammatory factors to infiltrate the CNS and activate hippocampal astrocytes, which facilitated Lcn2 expression, neuroinflammation, and neuronal demise, ultimately instigating depressive‐like conduct. Nonetheless, nicotine treatment limited the activation of astrocytes and alleviated neuronal death, which may ameliorate the depressive symptoms induced by exposure to CS.

To simulate the chronic exposure encountered by workers, the experimental animals were subjected to multiple nasal instillations of silica suspension.^30^ Our previous study observed that mice exposed to coal dust repeatedly on multiple occasions exhibited a significant decrease in mobility and physical activity during the OFT after 28 days.^31^ Silica particles, in contrast to coal dust particles, promptly triggers of depression‐like behavior upon exposure. Administration of nicotine via drinking water provides a stress‐free route for chronic drug treatment and has been used in various experiments, such as for studying addiction and toxicity.^32^ At 200 μg/mL nicotine, the blood plasma cotinine levels can reach 100–200 ng/mL, which is similar to the levels of a moderate smoker.^33^ A relatively high dose of nicotine achieved the expected biological effect in the mouse brain. However, when the nicotine is taken away, it can cause withdrawal symptom.^34^

There is a close relationship between neuroinflammation and destruction of the BBB. Inhalation of multi‐walled carbon nanotubes can induce neuroinflammatory responses, which are associated with the disruption of the blood–brain barrier.^35^ The inhalation of 2.5 mg/kg of CS particles elicited an elevation of the pro‐inflammatory cytokines IL‐1β and IL‐6 within the lung, as well as a notable surge in pro‐inflammatory cytokines within the hippocampus, leading to synaptic impairment, amyloid‐β peptides aggregation, and compromised memory.^6^ After analyzing the effect of lung damage or systemic inflammation caused by CS inhalation, we focused on how this could impact neurodevelopmental and mood disorders associated with the hippocampus. Inhalable silica dust leads to the activation of circulating inflammatory factors such as IL‐6, which caused BBB damage and albumin leakage in this study. This indicated a tight link between peripheral inflammatory factors and secondary inflammation of the hippocampus accompanied by BBB structural deterioration. Drugs and inflammatory factors enhance the permeability of the blood–brain barrier (BBB), and, more significantly, the presence of inflammatory factors in the bloodstream disrupts BBB integrity, thereby contributing to immune cell‐mediated inflammation that affects brain tissue function.^36^ Targeting AQP4 as a specific modulator of BBB can mitigate post‐hemorrhagic edema (PHE) in intracerebral hemorrhage (ICH).^37^ Nicotine exhibited the potential to attenuate glial cell activation and uphold the integrity of the BBB.^38^ The data from our study revealed that exposure to CS resulted in an upregulation of AQP4 gene expression, which is one of the most abundant molecules located at the interfaces between the blood–brain barrier, following nicotine administration.

The mechanism of astrocyte activation induced by particulate matter is related to oxidative stress and cell inflammation, which eventually lead to central nervous system dysfunction.^39^ Recent studies have shown that mice prenatally exposed to PM2.5 resulted in a significant increase of astrocyte numbers within the hippocampal CA1 region, and activation of hippocampal astrocytes in response to nanoparticle exposure was closely related to the dosage.^40^, ^41^ It is widely acknowledged that astrocyte activation is a crucial factor in neuroinflammation, which in turn leads to neuronal damage.^42^ Recent research has revealed that reactive astrocytes can harm the nervous system by releasing toxins such as TNF‐α, IL‐1β, IL‐6, and saturated lipids, leading to synaptic abnormalities and neuronal death.^43^ However, some research has indicated that type 2 astrocytes (A2) can protect neurons by releasing neurotrophic factors, such as BDNF. Our study shows that CS exposure is associated with substantial neuronal death and neuroinflammation in the CA1 region of the hippocampus, highlighted by the upregulation of pro‐apoptotic markers such as TUNEL and BAX. Similar observations have been made in mice exposed to PM2.5.^44^ In the present study, CS entry into the body triggered astrocyte activation. However, nicotine exerted a neuroprotective effect by inhibiting astrocyte activation. Previous reports have demonstrated that nicotine can induce the release of neurotrophic factors from glial cells while inhibiting the expression of TNF‐α and ameliorating the activation of glial cells to a pro‐inflammatory state.^45^ Nicotine can increase both the volume and number of astrocytes and the concentration of neurotransmitters in the synaptic cleft, affecting the intracellular calcium ion concentration, extracellular matrix structure and synaptic plasticity of neurons.^46^ In ischemic rats, nicotine has been shown to improve cognitive ability by upregulating α4/β2‐nAChRs in nerve cells and suppressing neuroinflammation.^47^

Excessive upregulation of Lcn2 is commonly associated with astrocyte activation, which ultimately leads to the loss of BBB integrity and neuronal death.^48^ Recent experiments indicate that lipopolysaccharides (LPS), acting as inflammatory stressors, stimulate NF‐κB activation in reactive astrocytes, resulting in the secretion of Lcn2 and apoptosis in primary cultured neurons and causing neuroinflammation and emotional abnormality.^48^, ^49^ The nerve growth factor receptor can induce a neurogenic state in reactive astroglia by inhibiting the activity of Lcn2 on the Slc22a17 receptor, which protects neurons and suppresses neuroinflammation in AD mice.^50^ Inhibition of NF‐κB can reduce the expression of pro‐inflammatory cytokines (such as IL‐6, IL‐1β, and TNF‐α) and increase the expression of BDNF, thereby ameliorating the cognitive decline of diabetic mice.^51^ The involvement of Lcn2 in apoptosis extends to insulin resistance, cancer, and nervous system diseases.^52^, ^53^ However, further investigation is required to determine its potential role in particle‐induced conditions. Our findings that CS exposure augmented astrocytic Lcn2 and neurotoxicity in the hippocampus are in agreement with the bulk of the research.^19^, ^49^, ^54^ PM2.5 inhalation activates astrocytes and leads to neurotoxicity via the NF‐κB signaling pathway and oxidative stress.^55^ The NOX‐NF‐κB signaling pathway induces the upregulation of LCN2 expression in astrocytes within the ischemic brain, which results in neuronal apoptosis and axonal degeneration.^49^ According to Hasel et al., reactive astrocytes in the cortex are characterized by inflammatory markers such as Timp1, Gap43, Hspb1, Lcn2, and GFAP, as well as antigen‐presenting genes such as H2‐K1, H2‐T23, and H2‐D1.^56^ Our study classified these Lcn2^+^ astrocytes in the CS‐injured hippocampus as having a pro‐inflammatory role, expressing NF‐κB, and thereby promoting neuroinflammation, which can eventually lead to neuronal death. Additionally, nicotine has the effect of reducing Lcn2 levels, potentially shielding the hippocampus from CS‐induced harm. Nevertheless, Kang et al. demonstrated that Lcn2 can shield against brain injury in neuroinflammation.^57^ Further research is necessary to explore the subpopulations of astrocytes in different regions of the hippocampus, beyond the Lcn2 marker in astrocytes.^56^

The analysis of the GSEA database revealed that nicotine could downregulate the expression of inflammation‐ and apoptosis‐related genes, including Lcn2, Plin4, Scgb3a1, Lrg1, FAS, S100A8/A9, Ly6a, and Chil3 in the hippocampus following CS‐induced injury. The genes Lcn2, Plin4, Scgb3a1, and Lrg1 have been shown to cooperatively promote neuroinflammation. S100a8/a9, a calcium‐binding protein, is known to regulate the migration and activation of macrophages and inflammatory cells during inflammatory response.^58^ Ly6a and Chil3 are responsible for the infiltration of circulating lymphocytes, while Chil3 can also influence the occurrence and progression of neuroinflammation.^59^ Additionally, FAS, a membrane receptor protein, has been demonstrated to induce neuronal apoptosis.^60^ Following exposure to CS treatment, there is a significant upregulation in the expression of these factors, suggesting that silicon particles can cause a heightened neuroinflammatory response. However, nicotine treatment attenuates this expression in this study.

Activated astrocytes and microglia form a “triads” structure when they phagocytose neurons. The number of triad structures may be a new marker of glial cell activation and neuronal death. Our results confirmed a marked increase in the expression of C1q and C3 after CS exposure. The complement cascade components C1q and C3 localize to neuronal synapses, thereby promoting the phagocytosis of neurons at microglial synapses.^61^ Microglia and astrocytes are mobilized to phagocytose and eliminate damaged neurons.^62^ Furthermore, the sustained activation of microglia leads to the release of pro‐inflammatory mediators, which subsequently trigger self‐activation in microglia.^63^ The chemotactic microglia and astrocytes segregate and phagocytose the damaged or apoptotic neuronal cells, forming numerous “triads.” The quantification of these triad structures can be utilized to evaluate the activation of glial cells and neurons demise.^64^, ^65^ CA1 pyramidal neurons are more vulnerable to damaging agents and inflammatory stimuli. Upon activation, astrocytes and microglia take on the role of swallowing and eliminating neurons. Our study demonstrates that the upregulation of Lcn2 expression results in hippocampal neuronal death, leading to microglial phagocytosis. The triad structure exerts a beneficial impact on impeding hippocampal inflammation. It promptly eradicates the origins of neuroinflammation while restricting inflammation propagation. However, exposure to CS results in the sustained upregulation of Lcn2 expression, facilitating chronic hippocampal neuronal damage and continued activation of glial cells with consequent release of inflammatory mediators, ultimately leading to decreased hippocampal neurons, and emotional disturbances. Astrocytes might be the primary cell type affected in the initial stages of mild cognitive impairment.^66^ Astrocytes, which monitor and transmit a large number of “dangerous” signals, can amplify the degree of neuroinflammation.^67^ The activation of astrocytes relies on the presence of microglia. The interaction of inflammatory signals between microglia and astrocytes can be enhanced by the activation of the microglial self‐feedback loop and the unique anatomical structure of astrocytes within the immune network.^68^ However, the activation of microglia by astrocyte‐derived Lcn2 leads to neuroinflammation and subsequent indirect neurotoxicity.^19^

Nicotine exerts a neuroprotective effect in this regard. Nicotine improves depression‐induced hippocampal neuroplasticity by activating transmembrane ion channel receptor and the Notch signaling pathway and significantly alleviates depression and behavioral despair in stressed mice.^69^ Confocal localization of our immunofluorescence staining revealed that the NF‐κB increase mainly occurred in the nucleus of astrocytes, not in microglia (Figure 4). This suggests that the activation of NF‐κB in astrocytes may be involved in the development of neuroinflammation after CS exposure and that nicotine can inhibit it. NF‐κB is a key regulator of pro‐inflammatory mediators and is essential in innate and adaptive immune cells. Additionally, nicotine has been found to regulate the complex activity of monoamine oxidase and the electron transport chain, affording neuronal protection.^70^ Low doses of nicotine activate NAD^+^ salvage pathways and ameliorate age‐related issues in aged mice, such as increased energy metabolism and reduced inflammation.^71^ However, prolonged smoking or exposure to second‐hand smoke can have detrimental effects on neurons.^72^ The effects of different nicotine doses on astrocytes and microglia need to be further investigated in order to gain a better understanding of the underlying mechanisms involved in nicotine‐inhibited neuroinflammation. Our experiments showed that nicotine could protect CA1 neurons from dying and ease the depressed mood of mice 3 weeks after CS‐induced insult, yet this benefit diminished at weeks 6 and 9. Additional research is necessary to gain more insight into how chronic nicotine exposure affects the hippocampus when injured.

## 5 CONCLUSION

In conclusion, exposure to CS can significantly activate astrocytes and promote astrocyte‐derived Lcn2, which leads to neuronal death and drives depression‐like behavior in mice. In addition, nicotine exerts neuroprotective effects by inhibiting astrocyte activation in the hippocampus shortly after CS injury.

## AUTHOR CONTRIBUTIONS

Hangbing Cao: Experiment, Data analysis, Writing & Editing; Bing Li: Experiment, Data analysis, Writing & Editing; Min Mu: Data analysis; Shanshan Li: Review & Editing; Haoming Chen: Methodology; Huihui Tao: Review& Editing, Wenyang Wang: Review& Editing; Yuanjie Zou: Animal Experiment; Yehong Zhao: Data analysis; Yang Liu: Animal Experiment; Xinrong Tao: Supervision, Funding acquisition, designed this experiment and Review & Editing.

## FUNDING INFORMATION

This work was supported by the University Synergy Innovation Program of Anhui Province (GXXT‐2021‐077; GXXT‐2022‐065), The Clinical Medical Research Transformation Project of Anhui Province (202304295107020033), and The Open Research Grant of the Joint National‐Local Engineering Research Centre for Safe and Precise Coal Mining (EC2021008).

## CONFLICT OF INTEREST STATEMENT

The authors declare that they have no conflicts of interest.

## Supporting information


Data S1.

## Data Availability

The datasets supporting the conclusions of this study are included within the article. The data that support the findings of this study are openly available in “figshare” at https://figshare.com/s/54e555e861445837a7e9.

## References

[1] Wang C , Yang LS , Shi XH , Yang YF , Liu K , Liu RY . Depressive symptoms in aged Chinese patients with silicosis. Aging Ment Health. 2008;12:343‐348. 18728947 10.1080/13607860802120938

[2] Russo M , Lucifora C , Pucciarelli F , Piccoli B . Work hazards and workers' mental health: an investigation based on the fifth European working conditions survey. Med Lav. 2019;110:115‐129. 30990473 10.23749/mdl.v110i2.7640 PMC7809969

[3] Ergun D , Ergun R , Ergan B , Kurt OK . Occupational risk factors and the relationship of smoking with anxiety and depression. Turk Thorac J. 2018;19:77‐83. 29755811 10.5152/TurkThoracJ.2017.17055 PMC5937814

[4] Xiao X , Shang X , Zhai B , Zhang H , Zhang T . Nicotine alleviates chronic stress‐induced anxiety and depressive‐like behavior and hippocampal neuropathology via regulating autophagy signaling. Neurochem Int. 2018;114:58‐70. 29339018 10.1016/j.neuint.2018.01.004

[5] Fujiwara K , Ikeda Y , Murakami Y , et al. Aqueous flare and progression of visual field loss in patients with retinitis Pigmentosa. Invest Ophthalmol Vis Sci. 2020;61:26. 10.1167/iovs.61.8.26 PMC7425686 32692839

[6] Suman PR , Souza LS , Kincheski GC , et al. Lung inflammation induced by silica particles triggers hippocampal inflammation, synapse damage and memory impairment in mice. J Neuroinflammation. 2022;19:303. 36527099 10.1186/s12974-022-02662-0 PMC9756632

[7] Ronsmans S , Sorig Hougaard K , Nawrot TS , et al. The EXIMIOUS project‐mapping exposure‐induced immune effects: connecting the exposome and the immunome. Environ Epidemiol. 2022;6:e193. 35169671 10.1097/EE9.0000000000000193 PMC8835560

[8] Navarro KM , Butler CR , Fent K , et al. The wildland firefighter exposure and health effect (WFFEHE) study: rationale, design, and Methods of a repeated‐measures study. Ann Work Expo Health. 2022;66:714‐727. 34919119 10.1093/annweh/wxab117 PMC9203592

[9] Britzolaki A , Cronin CC , Flaherty PR , Rufo RL , Pitychoutis PM . Chronic but not acute pharmacological activation of SERCA induces behavioral and neurochemical effects in male and female mice. Behav Brain Res. 2021;399:112984. 33137400 10.1016/j.bbr.2020.112984 PMC7855138

[10] Muddapu VR , Dharshini SAP , Chakravarthy VS , Gromiha MM . Neurodegenerative diseases – Is metabolic deficiency the root cause? Front Neurosci. 2020;14:213. 32296300 10.3389/fnins.2020.00213 PMC7137637

[11] Hansen SN , Jorgensen JMB , Nyengaard JR , Lykkesfeldt J , Tveden‐Nyborg P . Early life vitamin C deficiency does not Alter morphology of hippocampal CA1 pyramidal neurons or markers of synaptic plasticity in a Guinea pig model. Nutrients. 2018;10:749. 29890692 10.3390/nu10060749 PMC6024653

[12] Shuto T , Kuroiwa M , Sotogaku N , et al. Obligatory roles of dopamine D1 receptors in the dentate gyrus in antidepressant actions of a selective serotonin reuptake inhibitor, fluoxetine. Mol Psychiatry. 2020;25:1229‐1244. 30531938 10.1038/s41380-018-0316-x PMC7244404

[13] Grist JJ , Marro B , Lane TE . Neutrophils and viral‐induced neurologic disease. Clin Immunol. 2018;189:52‐56. 27288312 10.1016/j.clim.2016.05.009 PMC5145788

[14] Vojvodic J , Mihajlovic G , Vojvodic P , et al. The impact of immunological factors on depression treatment – relation between antidepressants and immunomodulation agents. Open Access Maced J Med Sci. 2019;7:3064‐3069. 31850124 10.3889/oamjms.2019.779 PMC6910782

[15] Zhang H , Wang Y , He Y , et al. A1 astrocytes contribute to murine depression‐like behavior and cognitive dysfunction, which can be alleviated by IL‐10 or fluorocitrate treatment. J Neuroinflammation. 2020;17:200. 32611425 10.1186/s12974-020-01871-9 PMC7331266

[16] Balta D , Zunke F . The role of lysosomes in alpha‐synucleinopathies: a focus on glial cells. Neural Regen Res. 2022;17:1486‐1488. 34916427 10.4103/1673-5374.330608 PMC8771110

[17] Zhao N , Xu X , Jiang Y , et al. Lipocalin‐2 may produce damaging effect after cerebral ischemia by inducing astrocytes classical activation. J Neuroinflammation. 2019;16:168. 31426811 10.1186/s12974-019-1556-7 PMC6699078

[18] Liu Y , Fu H , Wang T . Neuroinflammation in perioperative neurocognitive disorders: from bench to the bedside. CNS Neurosci Ther. 2022;28:484‐496. 34990087 10.1111/cns.13794 PMC8928922

[19] Kim JH , Ko PW , Lee HW , et al. Astrocyte‐derived lipocalin‐2 mediates hippocampal damage and cognitive deficits in experimental models of vascular dementia. Glia. 2017;65:1471‐1490. 28581123 10.1002/glia.23174

[20] Twyman L , Cowles C , Walsberger SC , Baker AL , Bonevski B , Tackling tobacco mental health advisory G . They're going to smoke Anyway': a qualitative study of community mental health staff and consumer perspectives on the role of social and living environments in tobacco use and cessation. Front Psych. 2019;10:503. 10.3389/fpsyt.2019.00503 PMC6652148 31379622

[21] Mineur YS , Einstein EB , Seymour PA , et al. alpha4beta2 nicotinic acetylcholine receptor partial agonists with low intrinsic efficacy have antidepressant‐like properties. Behav Pharmacol. 2011;22:291‐299. 21566524 10.1097/FBP.0b013e328347546d PMC3227135

[22] Letsinger AC , Ward JM , Fannin RD , et al. Nicotine exposure decreases likelihood of SARS‐CoV‐2 RNA expression and neuropathology in the hACE2 mouse brain but not moribundity. Res Sq. 2022;13:2042. 10.1038/s41598-023-29118-6 PMC9898857 36739463

[23] Park HJ , Lee PH , Ahn YW , et al. Neuroprotective effect of nicotine on dopaminergic neurons by anti‐inflammatory action. Eur J Neurosci. 2007;26:79‐89. 17581257 10.1111/j.1460-9568.2007.05636.x

[24] Radu M , Chernoff J . An in vivo assay to test blood vessel permeability. J Vis Exp. 2013;73:1940‐087X. 10.3791/50062 PMC3639515 23524912

[25] Song Q , Huang W , Ye W , et al. Neuroprotective effects of estrogen through BDNF‐transient receptor potential channels 6 signaling pathway in the hippocampus in a rat model of Perimenopausal depression. Front Aging Neurosci. 2022;14:14. 10.3389/fnagi.2022.869274 PMC9305198 35875795

[26] Tavares G , Martins M , Correia JS , et al. Employing an open‐source tool to assess astrocyte tridimensional structure. Brain Struct Funct. 2017;222:1989‐1999. 27696155 10.1007/s00429-016-1316-8 PMC5406431

[27] Schindelin J , Arganda‐Carreras I , Frise E , et al. Fiji: an open‐source platform for biological‐image analysis. Nat Methods. 2012;9:676‐682. 22743772 10.1038/nmeth.2019 PMC3855844

[28] Borroni E , Pesatori AC , Bollati V , Buoli M , Carugno M . Air pollution exposure and depression: a comprehensive updated systematic review and meta‐analysis. Environ Pollut. 2022;292:118245. 34600062 10.1016/j.envpol.2021.118245

[29] Picciotto MR , Lewis AS , van Schalkwyk GI , Mineur YS . Mood and anxiety regulation by nicotinic acetylcholine receptors: a potential pathway to modulate aggression and related behavioral states. Neuropharmacology. 2015;96:235‐243. 25582289 10.1016/j.neuropharm.2014.12.028 PMC4486625

[30] Chen H , Tao X , Cao H , et al. Nicotine exposure exacerbates silica‐induced pulmonary fibrosis via STAT3‐BDNF‐TrkB‐mediated epithelial‐mesenchymal transition in alveolar type II cells. Food Chem Toxicol. 2023;113694:113694. 10.1016/j.fct.2023.113694 36868510

[31] Zou Y , Mu M , Zhang S , et al. Vitamin D3 suppresses astrocyte activation and ameliorates coal dust‐induced mood disorders in mice. J Affect Disord. 2022;303:138‐147. 35157949 10.1016/j.jad.2022.02.026

[32] Zhou L , Tao X , Pang G , et al. Maternal nicotine exposure alters hippocampal microglia polarization and promotes anti‐inflammatory signaling in juvenile offspring in mice. Front Pharmacol. 2021;12:661304. 34045967 10.3389/fphar.2021.661304 PMC8144443

[33] Zhao‐Shea R , DeGroot SR , Liu L , et al. Increased CRF signalling in a ventral tegmental area‐interpeduncular nucleus‐medial habenula circuit induces anxiety during nicotine withdrawal. Nat Commun. 2015;6:6770. 25898242 10.1038/ncomms7770 PMC4405813

[34] Lawson GM , Hurt RD , Dale LC , et al. Application of serum nicotine and plasma cotinine concentrations to assessment of nicotine replacement in light, moderate, and heavy smokers undergoing transdermal therapy. J Clin Pharmacol. 1998;38(6):502‐509. 9650539 10.1002/j.1552-4604.1998.tb05787.x

[35] Aragon MJ , Topper L , Tyler CR , et al. Serum‐borne bioactivity caused by pulmonary multiwalled carbon nanotubes induces neuroinflammation via blood‐brain barrier impairment. Proc Natl Acad Sci U S A. 2017;114:E1968‐E1976. 28223486 10.1073/pnas.1616070114 PMC5347541

[36] Zhang Y , Li Y , Han Y , et al. Experimental study of EGFR‐TKI aumolertinib combined with ionizing radiation in EGFR mutated NSCLC brain metastases tumor. Eur J Pharmacol. 2023;945:175571. 36804545 10.1016/j.ejphar.2023.175571

[37] Jeon H , Kim M , Park W , et al. Upregulation of AQP4 improves blood–brain barrier integrity and Perihematomal edema following intracerebral hemorrhage. Neurotherapeutics. 2021;18:2692‐2706. 34545550 10.1007/s13311-021-01126-2 PMC8804112

[38] Elahy M , Lam V , Pallebage‐Gamarallage MM , Giles C , Mamo JC , Takechi R . Nicotine attenuates disruption of blood‐brain barrier induced by saturated‐fat feeding in wild‐type mice. Nicotine Tob Res. 2015;17:1436‐1441. 25744960 10.1093/ntr/ntv044

[39] Li S , Fang Y , Zhang Y , et al. Microglial NLRP3 inflammasome activates neurotoxic astrocytes in depression‐like mice. Cell Rep. 2022;41:41. 10.1016/j.celrep.2022.111532 36288697

[40] Onoda A , Takeda K , Umezawa M . Dose‐dependent induction of astrocyte activation and reactive astrogliosis in mouse brain following maternal exposure to carbon black nanoparticle. Part Fibre Toxicol. 2017;14:4. 28148272 10.1186/s12989-017-0184-6 PMC5289048

[41] Di Domenico M , Benevenuto SGM , Tomasini PP , et al. Concentrated ambient fine particulate matter (PM2.5) exposure induce brain damage in pre and postnatal exposed mice. Neurotoxicology. 2020;79:127‐141. 32450181 10.1016/j.neuro.2020.05.004

[42] Absinta M , Maric D , Gharagozloo M , et al. A lymphocyte‐microglia‐astrocyte axis in chronic active multiple sclerosis. Nature. 2021;597:709‐714. 34497421 10.1038/s41586-021-03892-7 PMC8719282

[43] Jiang K , Sun Y , Chen X . Mechanism underlying acupuncture therapy in spinal cord injury: a narrative overview of preclinical studies. Front Pharmacol. 2022;13:875103. 35462893 10.3389/fphar.2022.875103 PMC9021644

[44] Chuang HC , Chen HC , Chai PJ , et al. Neuropathology changed by 3‐ and 6‐months low‐level PM2.5 inhalation exposure in spontaneously hypertensive rats. Part Fibre Toxicol. 2020;17:59. 33243264 10.1186/s12989-020-00388-6 PMC7691081

[45] Nicholatos JW , Francisco AB , Bender CA , et al. Nicotine promotes neuron survival and partially protects from Parkinson's disease by suppressing SIRT6. Acta Neuropathol Commun. 2018;6:120. 30409187 10.1186/s40478-018-0625-y PMC6223043

[46] Aryal SP , Fu X , Sandin JN , et al. Nicotine induces morphological and functional changes in astrocytes via nicotinic receptor activity. Glia. 2021;69:2037‐2053. 33851731 10.1002/glia.24011 PMC8258843

[47] Gou J , Liang S , Cheng W , et al. Neuroprotective effect of combined use of nicotine and celecoxib by inhibiting neuroinflammation in ischemic rats. Brain Res Bull. 2021;175:234‐243. 34333049 10.1016/j.brainresbull.2021.07.022

[48] Chen X , Qiu F , Zhao X , et al. Astrocyte‐derived Lipocalin‐2 Is involved in mitochondrion‐related neuronal apoptosis induced by methamphetamine. ACS Chem Nerosci. 2020;11:1102‐1116. 10.1021/acschemneuro.9b00559 32186847

[49] Liu R , Wang J , Chen Y , et al. NOX activation in reactive astrocytes regulates astrocytic LCN2 expression and neurodegeneration. Cell Death Dis. 2022;13:371. 35440572 10.1038/s41419-022-04831-8 PMC9018876

[50] Siddiqui T , Cosacak MI , Popova S , et al. Nerve growth factor receptor (Ngfr) induces neurogenic plasticity by suppressing reactive astroglial Lcn2/Slc22a17 signaling in Alzheimer's disease. NPJ Regen Med. 2023;8:33. 37429840 10.1038/s41536-023-00311-5 PMC10333226

[51] Zhao B , Fei Y , Zhu J , Yin Q , Fang W , Li Y . PAF receptor inhibition attenuates neuronal Pyroptosis in cerebral ischemia/reperfusion injury. Mol Neurobiol. 2021;58:6520‐6539. 34562185 10.1007/s12035-021-02537-0

[52] Wu D , Wang X , Han Y , Wang Y . The effect of lipocalin‐2 (LCN2) on apoptosis: a proteomics analysis study in an LCN2 deficient mouse model. BMC Genomics. 2021;22:892. 34903175 10.1186/s12864-021-08211-y PMC8670060

[53] Xiang X , Tang X , Yu Y , et al. Role of lipocalin‐2 in surgery‐induced cognitive decline in mice: a signal from neuron to microglia. J Neuroinflammation. 2022;19:92. 35413913 10.1186/s12974-022-02455-5 PMC9006597

[54] Jung BK , Park Y , Yoon B , et al. Reduced secretion of LCN2 (lipocalin 2) from reactive astrocytes through autophagic and proteasomal regulation alleviates inflammatory stress and neuronal damage. Autophagy. 2023;19:1‐22. 36781380 10.1080/15548627.2023.2180202 PMC10351455

[55] Zhao N , Francis NL , Calvelli HR , Moghe PV . Microglia‐targeting nanotherapeutics for neurodegenerative diseases. APL Bioengineering. 2020;4:30902. 10.1063/5.0013178 PMC7481010 32923843

[56] Hasel P , Rose IVL , Sadick JS , Kim RD , Liddelow SA . Neuroinflammatory astrocyte subtypes in the mouse brain. Nat Neurosci. 2021;24:1475‐1487. 34413515 10.1038/s41593-021-00905-6

[57] Kang SS , Ren Y , Liu CC , et al. Lipocalin‐2 protects the brain during inflammatory conditions. Mol Psychiatry. 2018;23:344‐350. 28070126 10.1038/mp.2016.243 PMC5503822

[58] Tappeiner C , Klotsche J , Sengler C , et al. Risk factors and biomarkers for the occurrence of uveitis in juvenile idiopathic arthritis: data from the inception cohort of newly diagnosed patients with juvenile idiopathic arthritis study. Arthritis Rheumatol. 2018;70:1685‐1694. 29732713 10.1002/art.40544 PMC6174956

[59] Starossom SC , Campo Garcia J , Woelfle T , et al. Chi3l3 induces oligodendrogenesis in an experimental model of autoimmune neuroinflammation. Nat Commun. 2019;10:217. 30644388 10.1038/s41467-018-08140-7 PMC6333780

[60] Krzyzowska M , Kowalczyk A , Skulska K , Thorn K , Eriksson K . Fas/FasL contributes to HSV‐1 brain infection and Neuroinflammation. Front Immunol. 2021;12:714821. 34526992 10.3389/fimmu.2021.714821 PMC8437342

[61] Spiteri AG , Wishart CL , King NJC . Immovable object meets unstoppable force? Dialogue between resident and peripheral myeloid cells in the inflamed brain. Front Immunol. 2020;11:600822. 33363542 10.3389/fimmu.2020.600822 PMC7752943

[62] Zou Z , Li L , Schafer N , Huang Q , Maegele M , Gu Z . Endothelial glycocalyx in traumatic brain injury associated coagulopathy: potential mechanisms and impact. J Neuroinflammation. 2021;18:134. 34126995 10.1186/s12974-021-02192-1 PMC8204552

[63] De Kort AM , Kuiperij HB , Alcolea D , et al. Cerebrospinal fluid levels of the neurotrophic factor neuroleukin are increased in early Alzheimer's disease, but not in cerebral amyloid angiopathy. Alzheimers Res Ther. 2021;13:160. 34560885 10.1186/s13195-021-00899-0 PMC8464117

[64] Tarudji AW , Gee CC , Romereim SM , Convertine AJ , Kievit FM . Antioxidant thioether core‐crosslinked nanoparticles prevent the bilateral spread of secondary injury to protect spatial learning and memory in a controlled cortical impact mouse model of traumatic brain injury. Biomaterials. 2021;272:120766. 33819812 10.1016/j.biomaterials.2021.120766 PMC8068673

[65] Lana D , Ugolini F , Nosi D , Wenk GL , Giovannini MG . The emerging role of the interplay among astrocytes, microglia, and neurons in the hippocampus in health and disease. Front Aging Neurosci. 2021;13:651973. 33889084 10.3389/fnagi.2021.651973 PMC8055856

[66] Polanco JC , Li C , Bodea LG , Martinez‐Marmol R , Meunier FA , Gotz J . Amyloid‐beta and tau complexity – towards improved biomarkers and targeted therapies. Nat Rev Neurol. 2018;14:22‐39. 29242522 10.1038/nrneurol.2017.162

[67] Liu LR , Liu JC , Bao JS , Bai QQ , Wang GQ . Interaction of microglia and astrocytes in the neurovascular unit. Front Immunol. 2020;11:1024. 32733433 10.3389/fimmu.2020.01024 PMC7362712

[68] Yu X , Khakh BS . SnapShot: astrocyte interactions. Cell. 2022;185:220,e1. 34995516 10.1016/j.cell.2021.09.029

[69] Shang X , Shang Y , Fu J , Zhang T . Nicotine significantly improves chronic stress‐induced impairments of cognition and synaptic plasticity in mice. Mol Neurobiol. 2017;54:4644‐4658. 27405470 10.1007/s12035-016-0012-2

[70] Liu YHJ , Wu J , Zhu C , et al. α7 nicotinic acetylcholine receptor‐mediated neuroprotection against dopaminergic neuron loss in an MPTP mouse model via inhibition of astrocyte activation. J Neuroinflammation. 2012;9:98. 22624500 10.1186/1742-2094-9-98 PMC3416733

[71] Yang L , Shen J , Liu C , et al. Nicotine rebalances NAD(+) homeostasis and improves aging‐related symptoms in male mice by enhancing NAMPT activity. Nat Commun. 2023;14:900. 36797299 10.1038/s41467-023-36543-8 PMC9935903

[72] Doiron M , Dupre N , Langlois M , Provencher P , Simard M . Smoking history is associated to cognitive impairment in Parkinson's disease. Aging Ment Health. 2017;21:322‐326. 26416159 10.1080/13607863.2015.1090393

