# Supplementary material for: Nicotine suppresses crystalline silica‐induced astrocyte activation and neuronal death by inhibiting NF‐κB in the mouse hippocampus
Source: CNS Neurosci Ther. 2023 Oct 21;30(4):e14508. doi: 10.1111/cns.14508 (PMC11017465; doi:10.1111/cns.14508)
Supplement: Supplementary file 1 — Data S1. [file CNS-30-e14508-s001.zip › supplement.docx]

**
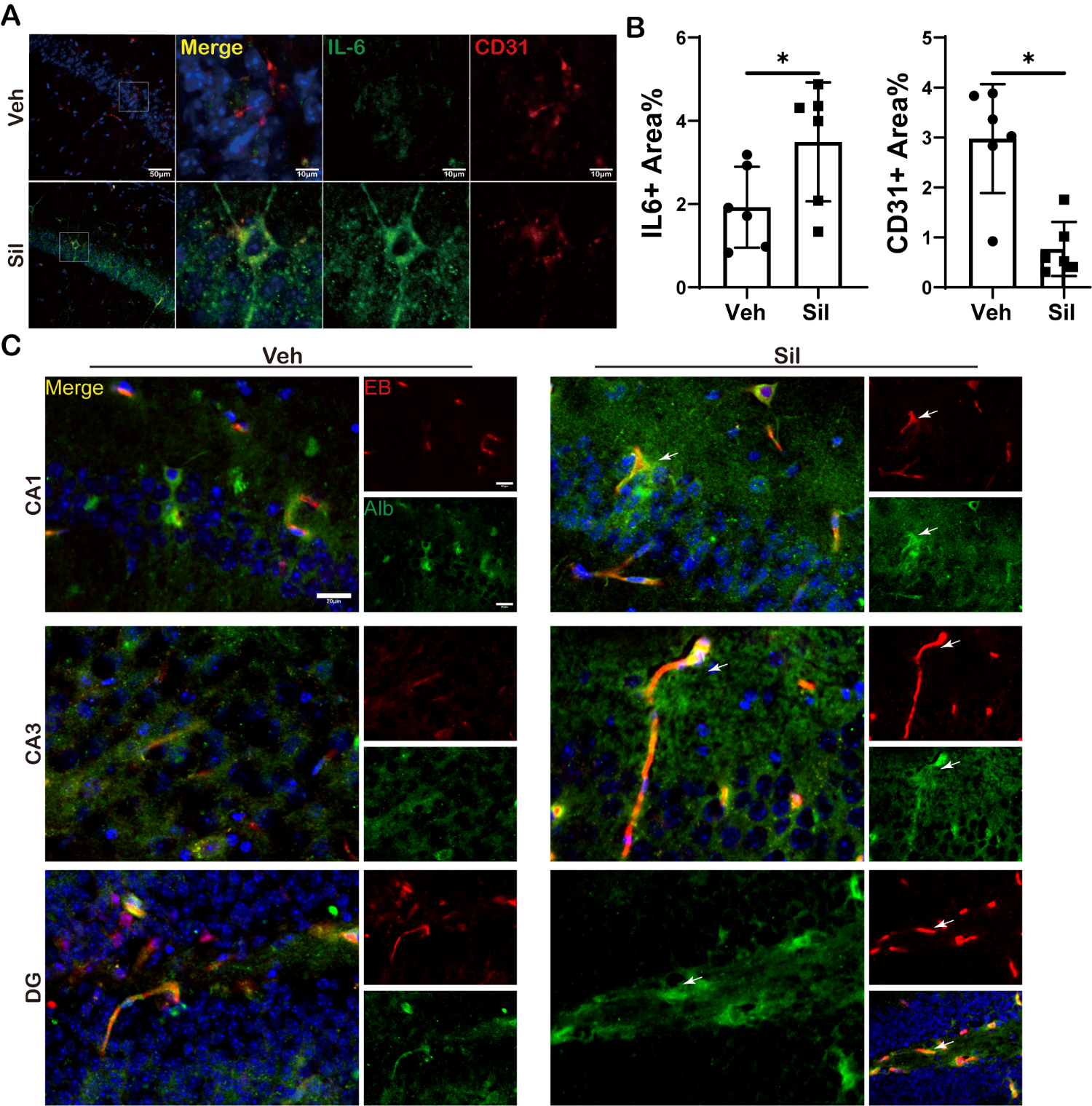
**

**Additional file 1: Fig. S1 CS exposure increased the damage of the BBB in mice.** **(A)** Representative graph showing IL-6 (green) and CD31 (red) in CA1. Scale bar: 50 μm and 10 μm. **(B,C)** Representative graph showing the BBB leakage. Immunofluorescent staining for Cldn-5 (green) and EB (red), Albumin (green), and EB (red) in CA1, CA3, and DG areas. Scale bar: 20 μm.


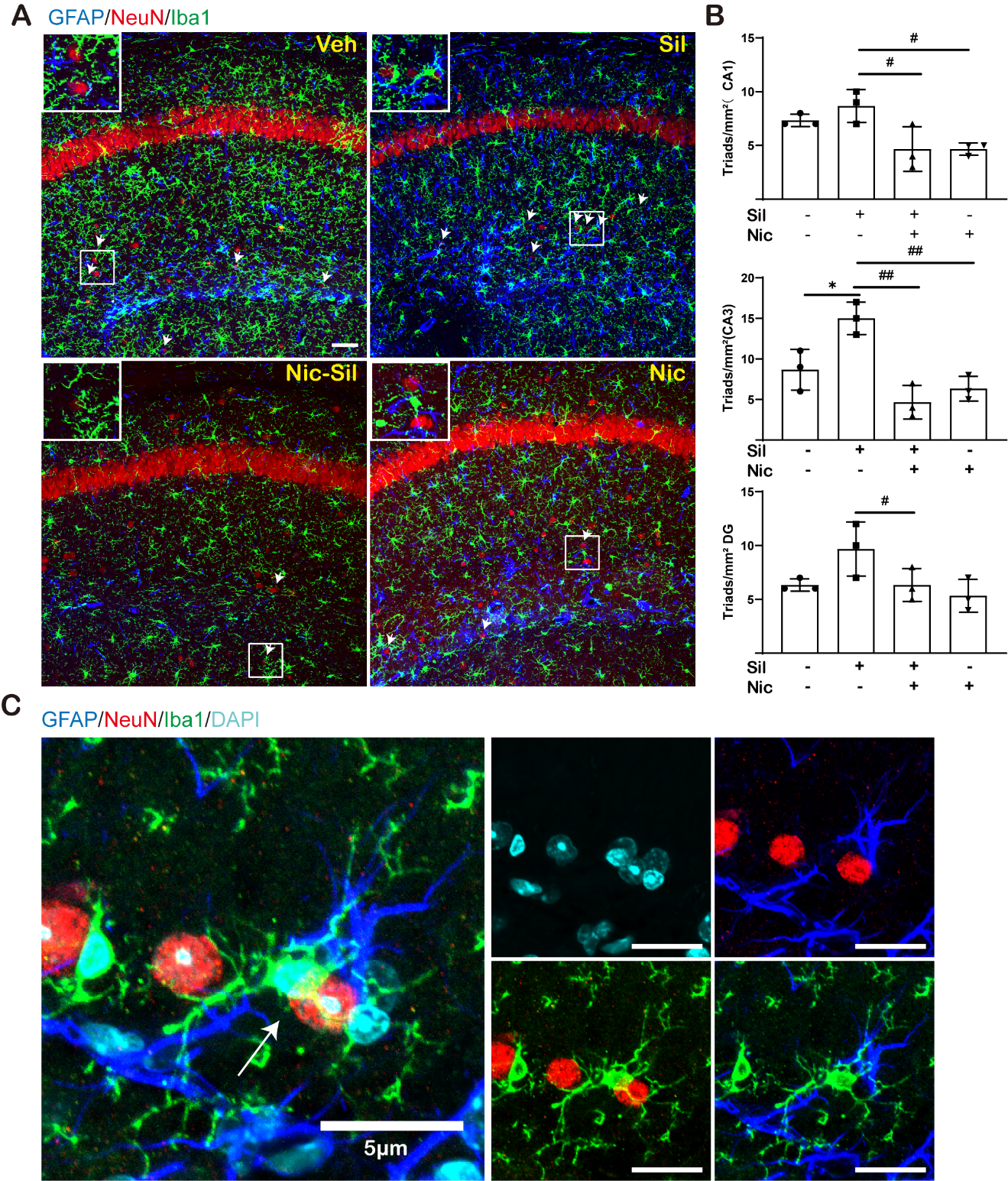


**Additional file 2: Fig. S2 Nicotine reduced the neuron-astrocyte-microglia "triads" in the CA1 region after CS exposure.** **(A)** Representative photomicrographs of triple immunostaining for neurons (red), microglia (green), astrocytes (blue), and DAPI (cyan blue) in CA1 of among Veh, Sil, Sil+Nic, and Nic group. White clippings indicate the structure of “triads”. Scale bar: 50 μm. **(B)** Quantitative statistics of “triads” in the CA1, CA3, and DG regions of the hippocampus, * *p* < 0.05 vs. Veh group; ^#^ *p* < 0.05, ^##^ *p* < 0.01 vs. Sil group. **(C)** Neuron-astrocyte-microglia "triads" enlargement showing triplex structure. n = 3 per experimental group, Scale bar: 5 μm.


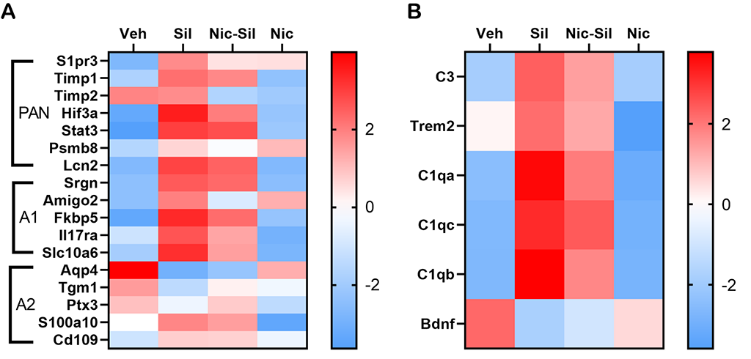


**Additional file 3: Fig. S3 A heat map revealing the difference expression genes connected to the glial-induced death of neurons.** **(A)** Astrocyte activation and **(B)** Neuronal death-related gene expression heat map. Screening conditions *p* < 0.05, Fold change > 1.2. n = 3 per experimental group.


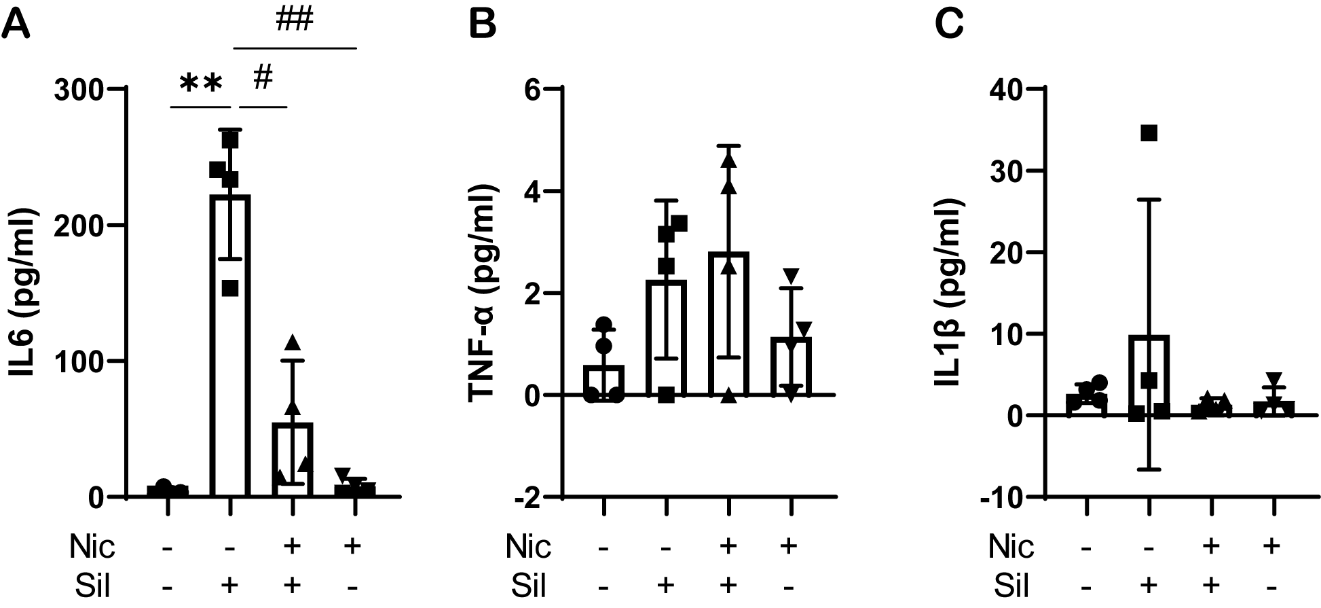


**Additional file 4: Fig. S4 Expression of inflammatory factors in the blood of mice at week 3 of the model.** **(A)** IL-6, **(B)** TNF-α, **(C)** IL-1β, n = 4 per experimental group, ** *p* < 0.01 *vs* Veh group; # *p* < 0.05, ## *p* < 0.01 *vs* Sil group.


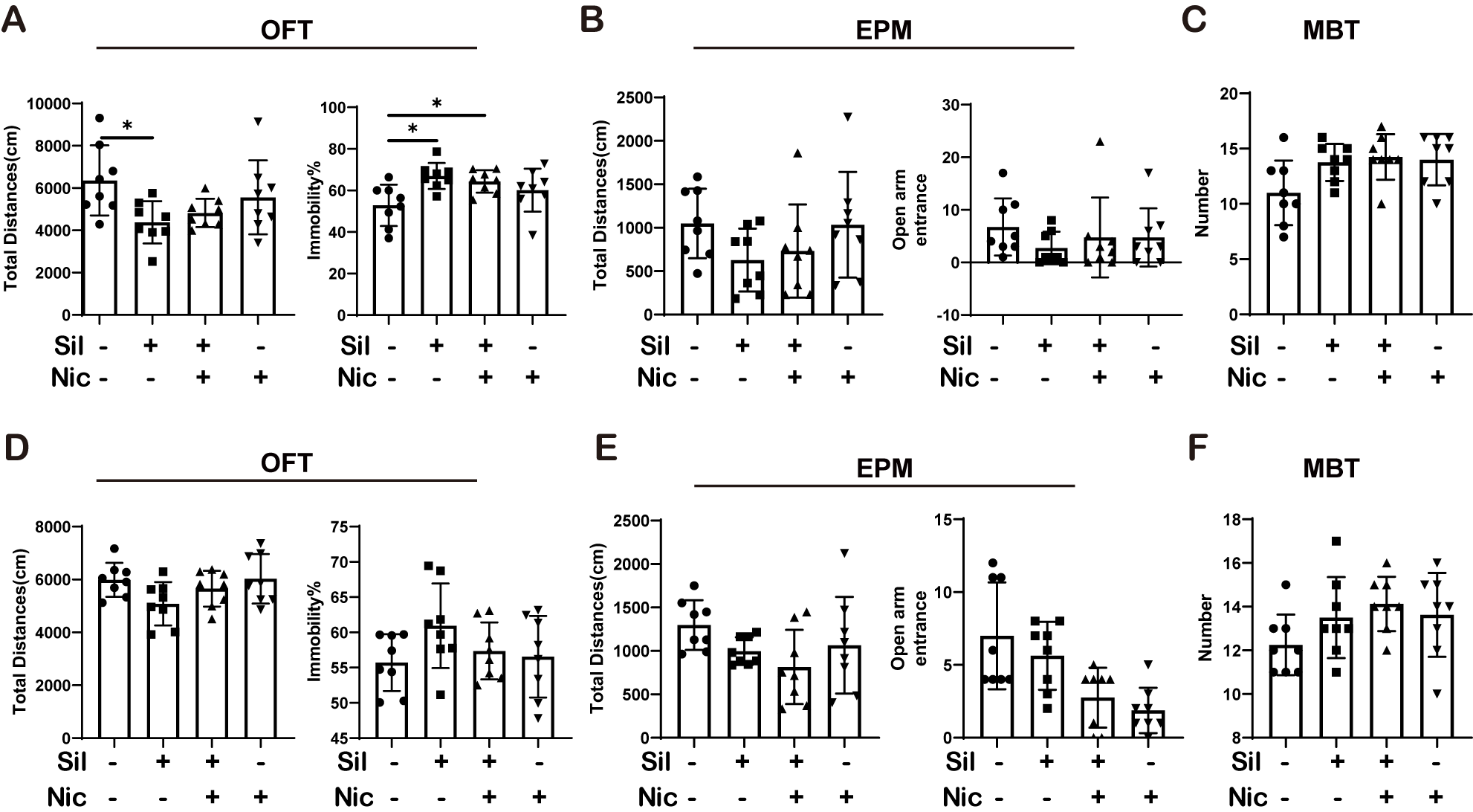


**Additional file 5: Fig. S5 Behavioral changes in mice at weeks 6 and 9.** **(A)** The total distances and immobility time (%) in the OFT at 6 weeks, * *p* < 0.05 *vs.* Veh group. **(B)** The total distances and open arm entrance in the EPM at 6 weeks. **(C)** The number of beads buried in the MBT at 6 weeks. **(D)** The total distances and immobility time (%) in the OFT at 9 weeks. **(E)** The total distances and open arm entrance in the EPM at 9 weeks. **(F)** The number of beads buried in the MBT at 9 weeks. n = 12 per experimental group.
