# Supplementary figures and images for: Nicotine suppresses crystalline silica‐induced astrocyte activation and neuronal death by inhibiting NF‐κB in the mouse hippocampus
Source: CNS Neurosci Ther. 2023 Oct 21;30(4):e14508. doi: 10.1111/cns.14508 (PMC11017465; doi:10.1111/cns.14508)

# Full unedited gel/blot for Figure 4

Veh      Sil      Nic+Sil      Nic

kDa

70  
55

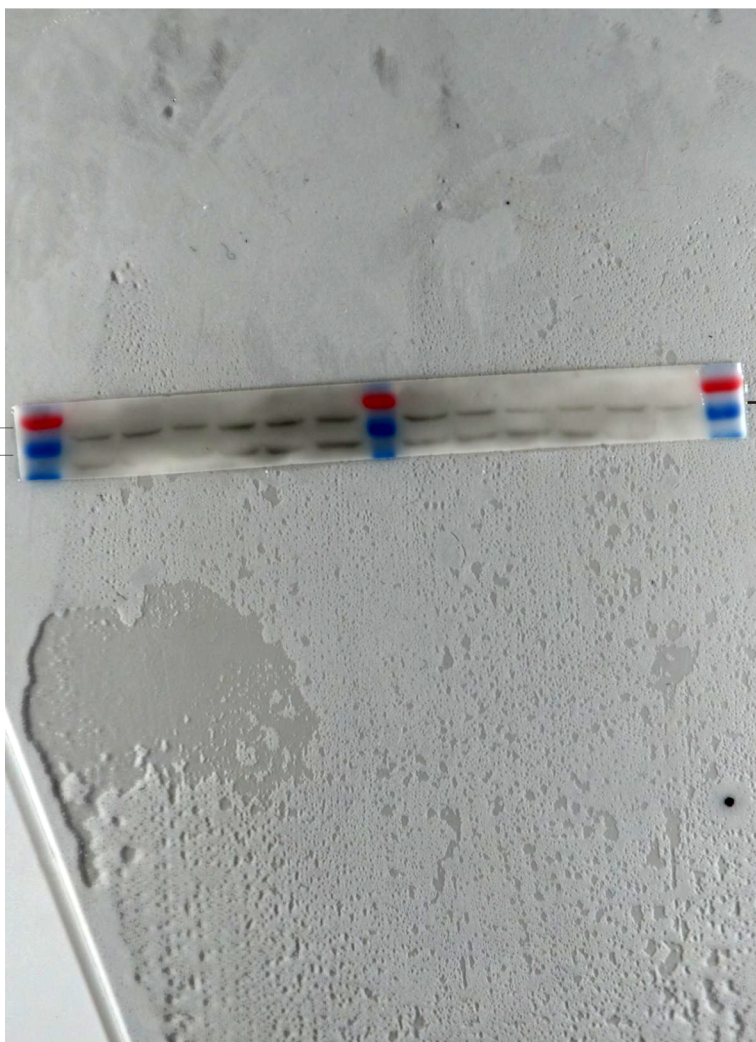

NF-κB p65

kDa

45  
35

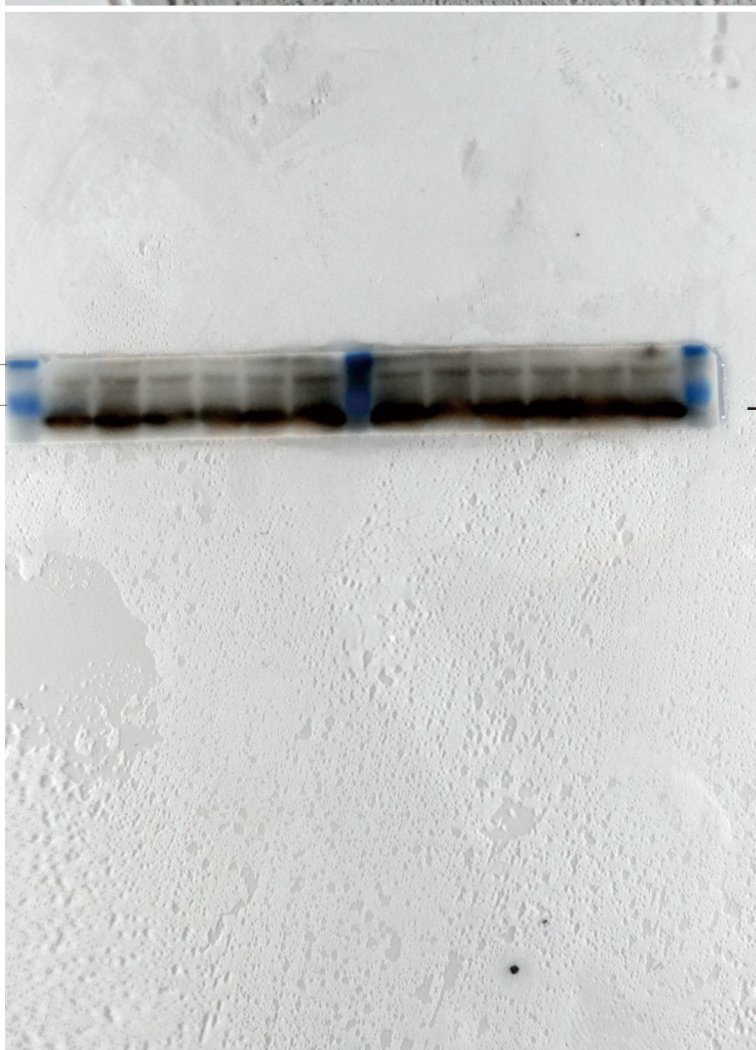

GAPDH

Supplement: Supplementary file 1 — Data S1. [file CNS-30-e14508-s001.zip › Full unedited blot for Figure 4.pdf]
